# Cryo-EM structures of PP2A:B55–FAM122A and PP2A:B55–ARPP19

**DOI:** 10.1038/s41586-023-06870-3

**Published:** 2023-12-20

**Authors:** Sathish K. R. Padi, Margaret R. Vos, Rachel J. Godek, James R. Fuller, Thomas Kruse, Jamin B. Hein, Jakob Nilsson, Matthew S. Kelker, Rebecca Page, Wolfgang Peti

**Affiliations:** 1 Department of Molecular Biology and Biophysics, University of Connecticut Health Center, Farmington, CT USA; 2 Department of Cell Biology, University of Connecticut Health Center, Farmington, CT USA; 3 Helix Biostructures, Indianapolis, IN USA; 4 The Novo Nordisk Foundation Center for Protein Research, University of Copenhagen, Copenhagen, Denmark

**Keywords:** Cryoelectron microscopy, Cell signalling, Enzyme mechanisms, Mitosis

## Abstract

Progression through the cell cycle is controlled by regulated and abrupt changes in phosphorylation^1^. Mitotic entry is initiated by increased phosphorylation of mitotic proteins, a process driven by kinases^2^, whereas mitotic exit is achieved by counteracting dephosphorylation, a process driven by phosphatases, especially PP2A:B55^3^. Although the role of kinases in mitotic entry is well established, recent data have shown that mitosis is only successfully initiated when the counterbalancing phosphatases are also inhibited^4^. Inhibition of PP2A:B55 is achieved by the intrinsically disordered proteins ARPP19^5,6^ and FAM122A^7^. Despite their critical roles in mitosis, the mechanisms by which they achieve PP2A:B55 inhibition is unknown. Here, we report the single-particle cryo-electron microscopy structures of PP2A:B55 bound to phosphorylated ARPP19 and FAM122A. Consistent with our complementary NMR spectroscopy studies, both intrinsically disordered proteins bind PP2A:B55, but do so in highly distinct manners, leveraging multiple distinct binding sites on B55. Our extensive structural, biophysical and biochemical data explain how substrates and inhibitors are recruited to PP2A:B55 and provide a molecular roadmap for the development of therapeutic interventions for PP2A:B55-related diseases.

## Abstract (web summary)

Cryo-electron microscopy structures of the PP2A:B55 holoenzyme bound to its inhibitors ARPP19 and FAM122A show distinct binding modes of the two inhibitors.

## Main

**Fig. 1:**
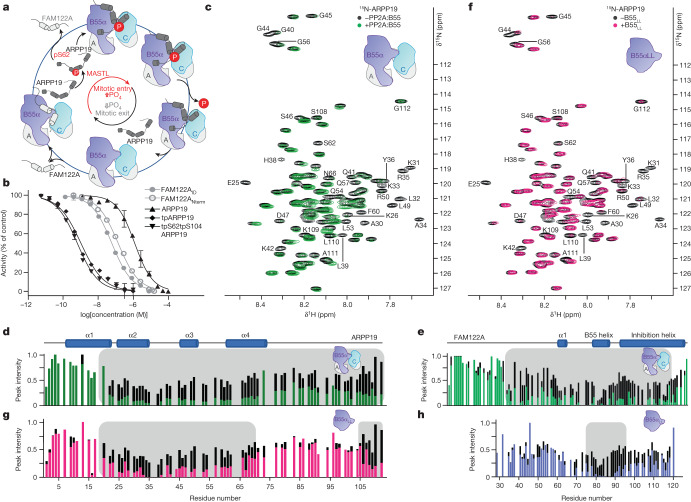
ARPP19 and FAM122A inhibit PP2A:B55. **a**, ARPP19 and FAM122A sequentially inhibit PP2A:B55 activity during mitosis. A, PP2Aa; C, PP2Ac; P, phosphate. **b**, PP2A:B55 inhibition by ARPP19 (with or without phosphorylation), FAM122A_Nterm_ and FAM122A_ID_ (mean ± s.d.; *n* = 3 experimental replicates). Results representative of *n* = 3 independent experiments. Unpaired two-tailed *t*-test with 95% confidence interval was used to compare ARPP19 with tpARPP19 (*P* < 0.0001) or tpS62tpS104ARPP19 (*P* < 0.0001). IC_50_ values are reported in Extended Data Table 1. **c**, 2D ^1^H,^15^N HSQC spectrum of ^15^N-labelled ARPP19 with or without PP2A:B55. **d**, Plot of peak intensity versus ARPP19 protein sequence for spectra in **c**; grey shading highlights ARPP19 residues with reduced intensities in the presence of PP2A:B55. Secondary structure elements based on NMR CSI data are indicated. Colour scheme as in **c**. **e**, Plot of peak intensity versus FAM122A protein sequence for FAM122A_Nterm_ alone (black) and with PP2A:B55 (green); grey shading highlights FAM122A residues with reduced intensities in the presence of PP2A:B55. Secondary structure elements based on NMR CSI data are indicated. **f**, 2D ^1^H,^15^N HSQC spectrum of ^15^N-labelled ARPP19 with (pink) and without (black) B55_LL_; grey shading highlights ARPP19 residues with reduced intensities in the presence of B55_LL_. **g**, Plot of peak intensity versus ARPP19 protein sequence for spectra in **f**; grey shading highlights ARPP19 residues with reduced intensities in the presence of B55_LL_. **h**, Plot of peak intensity versus FAM122A_ID_ protein sequence for FAM122A_ID_ alone (black) and with B55_LL_ (blue); grey shading highlights FAM122A_ID_ residues with reduced intensities in the presence of B55_LL_.

An essential function of the PP2A:B55 holoenzyme is the control of cell cycle progression through mitosis^4–6,8,9^. Mitotic entry is accomplished via the activation of the CDK1–cyclin B kinase, whose activity enables nuclear envelope breakdown, chromatin condensation and spindle formation^10,11^. Mitotic exit is initiated by cyclin B ubiquitination via the anaphase promoting complex, leading to its degradation^12^, an event accompanied by the dephosphorylation of mitotic substrates by protein phosphatases (PPPs), especially PP2A:B55^13^. Notably, inhibition of PP2A:B55 activity during mitotic onset has been shown to create the necessary dynamic feedback for robust mitotic substrate phosphorylation^14^. The PP2A:B55 holoenzyme also regulates the entry into mitosis at the G2/M checkpoint, as PP2A:B55 inhibition allows normal progression through the checkpoint^8,9^. These essential PP2A:B55-inhibition events are achieved by its interaction with two distinct intrinsically disordered protein (IDP) inhibitors, cAMP regulated phosphoprotein 19 (ARPP19) and family with sequence similarity 122A^6,15–17^ (FAM122A). The mechanisms by which these inhibitors block PP2A:B55 activity differ, as ARPP19 strictly requires phosphorylation by MASTL kinase to inhibit PP2A:B55^5,6^, whereas FAM122A inhibits PP2A:B55 in a phosphorylation-independent manner^7,8^. The current data suggest that these IDP inhibitors engage PP2A:B55 sequentially during mitotic entry (Fig. 1a). Specifically, PP2A:B55 is initially bound and inhibited by FAM122A. This inhibition results in the full activation of mitotic kinases, including MASTL, which phosphorylates ARPP19 on Ser62 (pS62-ARPP19). Through a currently unknown mechanism, pS62-ARPP19 displaces FAM122A from PP2A:B55. pS62-ARPP19 functions first as an inhibitor of PP2A:B55 but later becomes a substrate^18^. This dephosphorylation reactivates PP2A:B55 and enables progression through mitotic exit. Despite our understanding of the importance of PP2A:B55 in mitosis and knowing the identity of the IDP inhibitors that mediate PP2A:B55 inhibition during mitotic entry, we still lack a detailed understanding of how this inhibition is achieved at a molecular level.

## ARPP19 and FAM122A bind PP2A:B55

**Extended Data Fig. 1:**
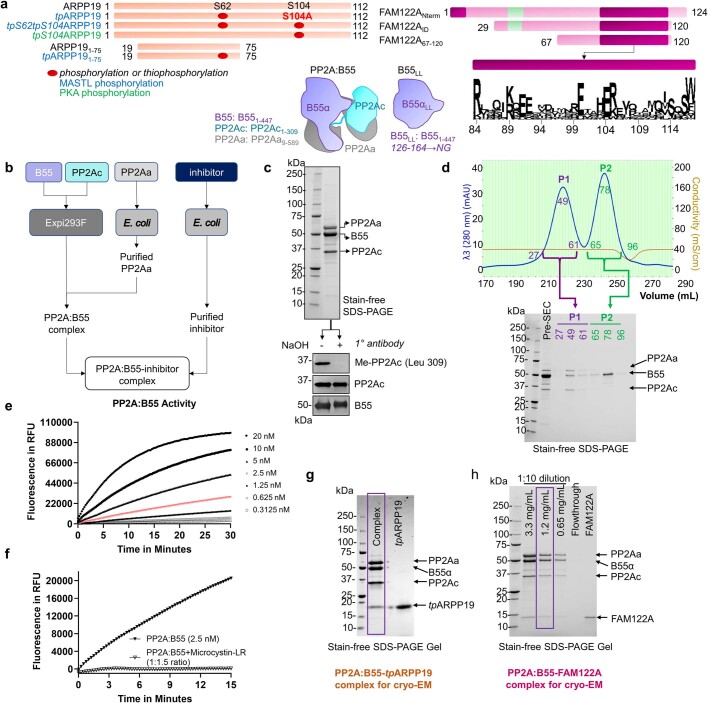
Constructs and complex production. **a**. Construct schematic. ARPP19 constructs phosphorylated as indicated. Most highly conserved FAM122A residues are shown. **b**. Schematic describing the production of PP2A:B55 and PP2A:B55-inhibitors for structural and biophysical studies. **c**. Immunoblots of purified PP2A:B55 (above, SDS-PAGE), with and without NaOH treatment, using antibodies detecting methylated PP2Ac (BioLegend, Cat# 828801), PP2Ac (Millipore, Cat# MABE1783) and B55 (Cell Signaling Technologies, Cat# 2290 S). **d**. Size exclusion chromatography (SEC) chromatogram of PP2A:B55; peak 1 corresponds to PP2A:B55; peak 2 is excess free B55. **e**. PP2A:B55 activity assay using DiFMUP as a substrate; PP2A:B55 concentrations 0.3125-20 nM; 2.5 nM concentration highlighted in red. **f**. Same as e (2.5 nM concentration) with and without the PPP inhibitor microcystin-LR (3.75 nM concentration). **g**. SDS-PAGE of the PP2A:B55-*tp*ARPP19 used for Cryo-EM grid preparation and data collection. **h**. SDS-PAGE of the PP2A:B55-FAM122A used for Cryo-EM grid preparation and data collection. Results (c-d and g-h) representative of 3 independent experiments.

**Extended Data Fig. 2:**
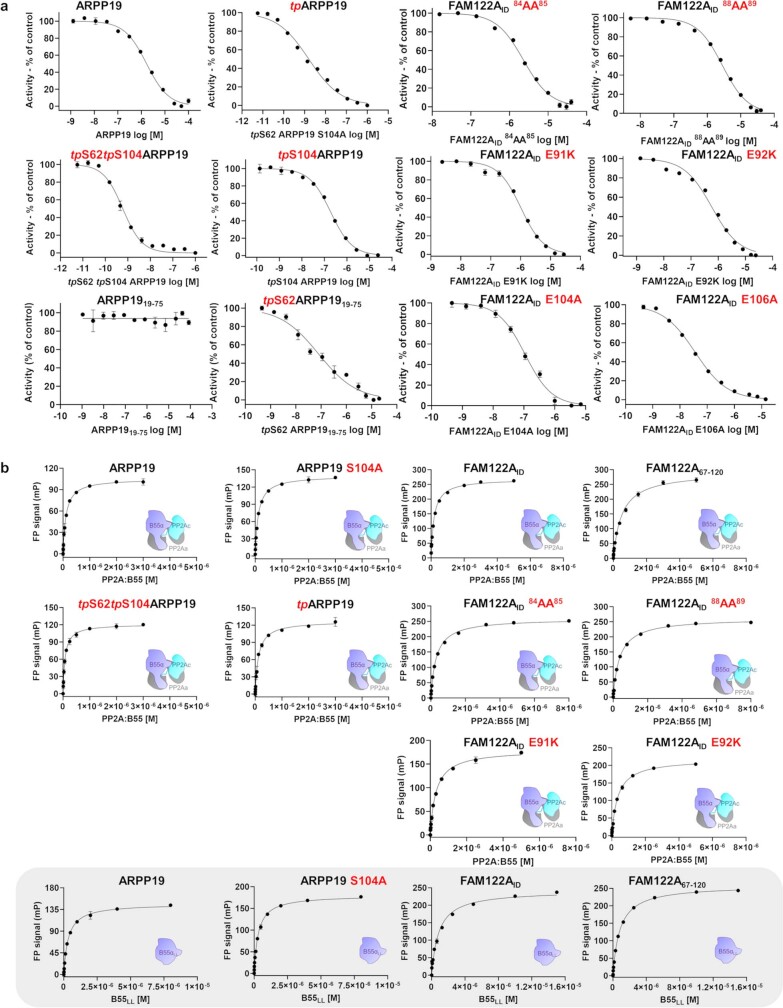
IC_50_ inhibition and binding assays for ARPP19 and FAM122A variants vs PP2A:B55 and B55_LL_. **a**. IC_50_ curves for PP2A:B55 inhibition by ARPP19 (variants; unphosphorylated and phosphorylated as well as different constructs) and FAM122A_ID_ variants. IC_50_ values are reported in Extended Data Table 1. Data are presented as mean values ± s.d. Results representative of n = 3 independent experiments. Unpaired two-tailed t-test with 95% confidence interval was used to compare: ARPP19 with *tp*ARPP19 (p < 0.0001) or *tpS62tpS104*ARPP19 (p < 0.0001) or *tp*ARPP19_19-75_ (p < 0.0001); *tp*ARPP19 with *tpS62tpS104*ARPP19 (p = 0.02) or *tp*ARPP19_19-75_ (p = 0.0002); FAM122A_ID_ with FAM122A_ID_ variants ^84^AA^85^ (p < 0.0001) or ^88^AA^89^ (p < 0.0001) or E91K (p < 0.0001) or E92K (p < 0.0001) or E104A (p < 0.0001) or E106A (p = 0.0013). **b**. FP binding studies to measure the interaction of ARPP19 (unphosphorylated and phosphorylated) and FAM122A_ID_ variants with PP2A:B55 and B55_LL_ (gray box). K_D_ values are reported in Extended Data Table 2. Data are presented as mean values ± s.d. Results representative of n = 3 independent experiments. Unpaired two-tailed t-test with 95% confidence interval was used to compare: PP2A:B55 interaction with ARPP19 vs *tpS62tpS104*ARPP19 (p = 0.0006); ARPP19 interaction with PP2A:B55 vs B55_LL_ (p = 0.0016); ARPP19 S104A interaction with PP2A:B55 vs B55_LL_ (p = 0.0008); PP2A:B55 interaction with FAM122A_ID_ vs FAM122A_67-120_ (p < 0.0001) or ^84^AA^85^ (p < 0.0001) or ^88^AA^89^ (p < 0.0001) or E91K (p < 0.0001) or E92K (p < 0.0001); FAM122A_ID_ interaction with PP2A:B55 vs B55_LL_ (p < 0.0001); FAM122A_67-120_ interaction with PP2A:B55 vs B55_LL_ (p < 0.0004).

**Extended Data Fig. 3:**
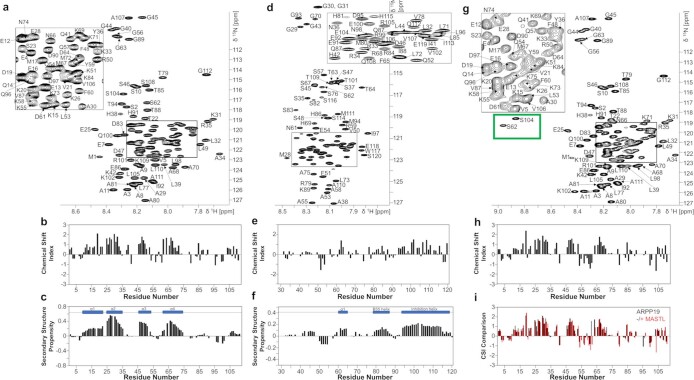
HSQC and CSI plots of ARPP19, FAM122A and MASTL-phosphorylated ARPP19. **a**. Fully annotated 2D [^1^H,^15^N] HSQC spectrum of ^15^N-labeled ARPP19. **b**. Chemical Shift Index (CSI) and **c**. Secondary-structure propensity (SSP) data for ARPP19 plotted vs. residue numbers. (SSP > 0, α helix; SSP < 0, β strand). Cα and Cβ chemical shifts were used to create the CSI and SSP plots (RefDB database^62^). Preferred secondary structure indicated above SSP data. **d**. Fully annotated 2D [^1^H,^15^N] HSQC spectrum of ^15^N-labeled FAM122A_ID_. **e**. CSI and **f**. SSP data for FAM122A_ID_ plotted vs. residue numbers; same as c. **g**. Fully annotated 2D [^1^H,^15^N] HSQC spectrum of ^15^N-labeled *tpS62tpS104*ARPP19. **h**. CSI; same as c. **i**. CSI comparison between ARPP19 (black) and *tpS62tpS104*ARPP19 (red).

**Extended Data Fig. 4:**
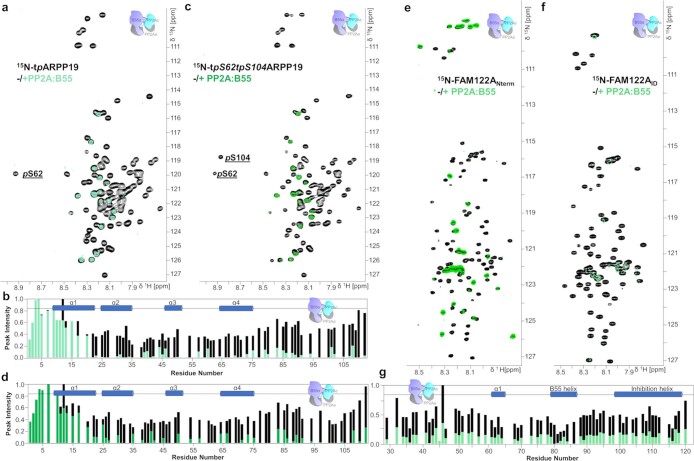
NMR data supporting the interaction of phosphorylated ARPP19 and FAM122A with PP2A:B55. **a**. 2D [^1^H,^15^N] HSQC spectrum of ^15^N-labeled *tp*ARPP19 alone (black) and in complex with PP2A:B55 (green). pS62 labeled for clarity. **b**. Peak intensity vs ARPP19 protein sequence plot for *tp*ARPP19 alone (black) and when bound to PP2A:B55 (green). Secondary structure elements based on NMR CSI data are indicated. **c**. 2D [^1^H,^15^N] HSQC spectrum of ^15^N-labeled *tpS62tpS104*ARPP19 alone (black) and in complex with PP2A:B55 (green). pS62 and pS104 labeled for clarity. **d**. Peak intensity vs ARPP19 protein sequence plot for *tpS62tpS104*ARPP19 alone (black) and when bound to PP2A:B55 (green). Secondary structure elements based on NMR CSI data are indicated. **e**. 2D [^1^H,^15^N] HSQC spectrum of ^15^N-labeled FAM122A_Nterm_ alone (black) and in complex with PP2A:B55 (green). **f**. 2D [^1^H,^15^N] HSQC spectrum of ^15^N-labeled FAM122A_ID_ alone (black) and in complex with PP2A:B55 (green). **g**. Peak intensity vs FAM122A_ID_ protein sequence plot for FAM122A_ID_ alone (black) and when bound to PP2A:B55 (green). Secondary structure elements based on NMR CSI data are indicated.

To determine how ARPP19 and FAM122A bind PP2A:B55, we established a method for producing high yields of active PP2A:B55 from Expi293F cells (Extended Data Fig. 1a–h); using this method, the C-terminal residue of PP2Ac is fully methylated^19,20^ (mLeu309). We quantified PP2A:B55 inhibition by ARPP19, thiophosphorylated ARPP19 (full-length (amino acids 1–112) and phosphorylated with ATPγS using MASTL kinase) and FAM122A (N-terminal domain (amino acids 1–124) (FAM122A_Nterm_)) (Fig. 1b). Whereas PP2A:B55 was only moderately inhibited by ARPP19, it was strongly inhibited by both thiophosphorylated ARPP19 and FAM122A_Nterm_ (Extended Data Table 1 and Extended Data Fig. 2a), with thiophosphorylated ARPP19 inhibiting PP2A:B55 around 250-fold more potently than FAM122A. Fluorescent polarization binding measurements showed that both FAM122A and ARPP19 bind PP2A:B55 tightly, with thiophosphorylation not influencing binding (Extended Data Table 2 and Extended Data Fig. 2b). We then used NMR spectroscopy to identify the residues in ARPP19 and FAM122A that interact with PP2A:B55. The 2D ^1^H,^15^N heteronuclear single quantum coherence (HSQC) spectra of unbound ARPP19^21,22^ and FAM122A_Nterm_ confirmed that both are IDPs with multiple regions of amino acids with preferred α-helical propensities (using chemical shift index (CSI) analysis; Extended Data Fig. 3a–f). Overlaying the 2D ^1^H,^15^N HSQC spectra of ARPP19 and FAM122A with and without PP2A:B55 identified the residues that bind PP2A:B55 (peaks with reduced intensities are due to either a direct interaction, a dynamic charge–charge interaction or conformational exchange on an intermediate timescale) (Fig. 1c–e). For ARPP19, around 90 N/H^N^ cross-peaks (residues 20–112) showed reduced intensities. Because ARPP19 inhibition of PP2A:B55 strictly requires phosphorylation, we also thiophosphorylated ARPP19 using MASTL kinase. The 2D ^1^H,^15^N HSQC spectrum of thiophosphorylated ARPP19 identified two phosphorylated residues, Ser62, the established MASTL phosphorylation substrate, and Ser104, a serine that was previously identified as a protein kinase A (PKA) substrate, and also shows recognition site homology to the MASTL specificity sequence^23^. The NMR data show that MASTL phosphorylation does not alter the preferred ensemble of structures of ARPP19 (Extended Data Fig. 3g–i). Thus, we generated the Ser104 phosphorylation site mutant ARPP19_S104A_ and repeated the thiophosphorylation step to obtain singly thiophosphorylated ARPP19 (tpS62ARPP19_S104A_; hereafter referred to as tpARPP19). Overlaying the 2D ^1^H,^15^N HSQC spectra of tpARPP19 and tpS62tpS104ARPP19 with PP2A:B55 showed that the intensities of the same approximately 90 N/H^N^ cross-peaks identified with unphosphorylated ARPP19 are reduced with PP2A:B55 (Extended Data Fig. 4a–d). Similar NMR interaction experiments with FAM122A_Nterm_ showed that the intensities of around 85 cross-peaks (residues 30–115) were reduced with PP2A:B55 (Fig. 1e and Extended Data Fig. 4e). On the basis of these data, we created FAM122A_ID_ (amino acids 29–120), which includes all PP2A:B55 interacting residues (Extended Data Figs. 3d–f and 4f,g).

**Extended Data Fig. 5:**
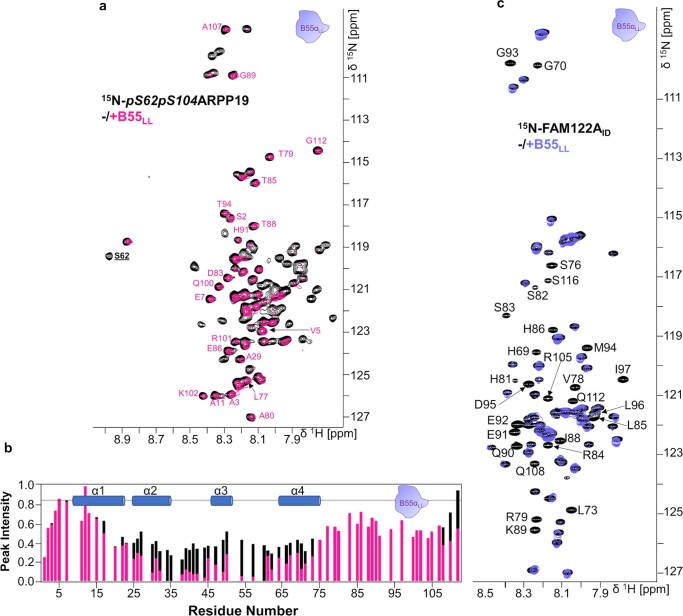
NMR data supporting the interaction of phosphorylated ARPP19 and FAM122A with B55_LL_. **a**. 2D [^1^H,^15^N] HSQC spectrum of ^15^N-labeled *pS62pS104*ARPP19 alone (black) and in complex with B55_LL_ (pink). pS62 labeled for clarity. **b**. Peak intensity vs ARPP19 protein sequence plot for *pS62pS104*ARPP19 alone (black) and when bound to B55_LL_ (pink). Secondary structure elements based on NMR CSI data are indicated. **c**. 2D [^1^H,^15^N] HSQC spectrum of ^15^N-labeled FAM122A_ID_ alone (black) and in complex with B55_LL_ (purple).

ARPP19 and FAM122A inhibit B55-containing PP2A holoenzymes^7,8^. To identify which residues of ARPP19 and FAM122A bind B55, we repeated the NMR experiments using B55 loopless (B55_LL_), a variant that lacks the PP2Aa binding loop (amino acids 126–164 are replaced with NG; Extended Data Fig. 1a) and is thus unable to bind PP2Aa. Overlaying the 2D ^1^H,^15^N HSQC spectra of ARPP19 and tpS62tpS104ARPP19 with and without B55_LL_ showed that the identity and number of N/H^N^ cross-peaks with reduced intensities are similar, but not identical, to those observed with PP2A:B55 (Fig. 1f,g and Extended Data Fig. 5a,b). Specifically, the peaks corresponding to residues 20–75 and 105–112 show significant reductions in intensities, whereas ARPP19 residues 75–104 show little or no intensity loss with B55_LL_. This shows that two distinct ARPP19 domains—20–75 and 105–112—bind B55. An overlay of the 2D ^1^H,^15^N HSQC spectrum of FAM122A_ID_ with and without B55_LL_ (Fig. 1h and Extended Data Fig. 5c) showed that N/H^N^ cross-peaks with reduced intensities correspond to FAM122A residues 73–95, which bind solely to B55. Both ARPP19 and FAM122A bind B55_LL_ with reduced affinities compared with PP2A:B55 (Extended Data Table 2 and Extended Data Fig. 2b). These B55 interaction regions of more than 20 residues were longer than expected (most PPPs, including PP2A:B56, bind their substrates and regulators using short linear motifs (SLIMs), that are typically 4–8 residues long, and bind their cognate PPP in an extended fashion^24–28^). This suggests that ARPP19 and FAM122A bind B55 via a different non-SLIM-based mechanism. Consistent with this, our NMR data showed that these IDP inhibitors exhibit helical propensities in solution (Extended Data Fig. 3c,f), suggesting they may bind B55 as helices.

## PP2A:B55–inhibitor cryo-EM structures

**Fig. 2:**
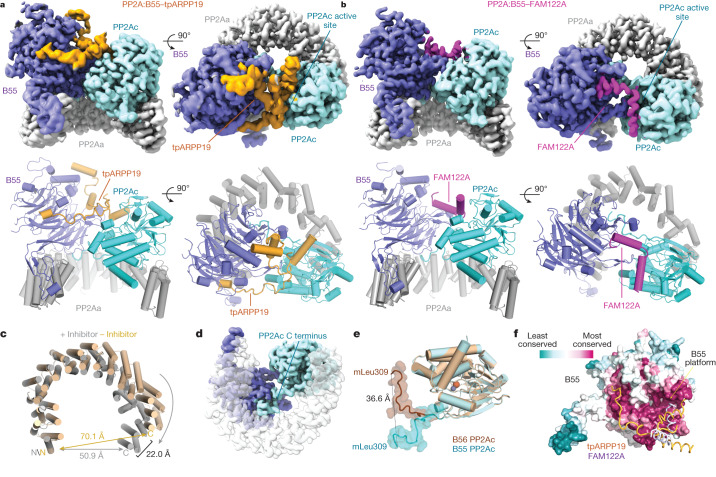
Structures of the PP2A:B55–tpARPP19 and PP2A:B55–FAM122A complexes. **a**, Cryo-EM map and model of PP2A:B55–tpARPP19. Two views of the map (top) with the corresponding view of the molecular model (bottom). **b**, Cryo-EM map and model of PP2A:B55–FAM122A. **c**, Overlay of PP2Aa from the PP2A:B55 crystal structure (PDB ID: 3DW8; beige) and the PP2A:B55–inhibitor model (grey), superimposed using HEAT repeats 1–6. **d**, Rotated view of the experimental map. **e**, Overlay of the PP2Ac catalytic domains from PP2A:B55-FAM122A (cyan) and PP2A:B56 (brown; PDB ID: 2IAE); the C-terminal tails (amino acids R294–mL309) shown as surfaces. **f**, B55 shown as surface and coloured by sequence conservation; ARPP19 (orange) and FAM122A (slate blue) are shown as ribbons.

**Extended Data Fig. 6:**
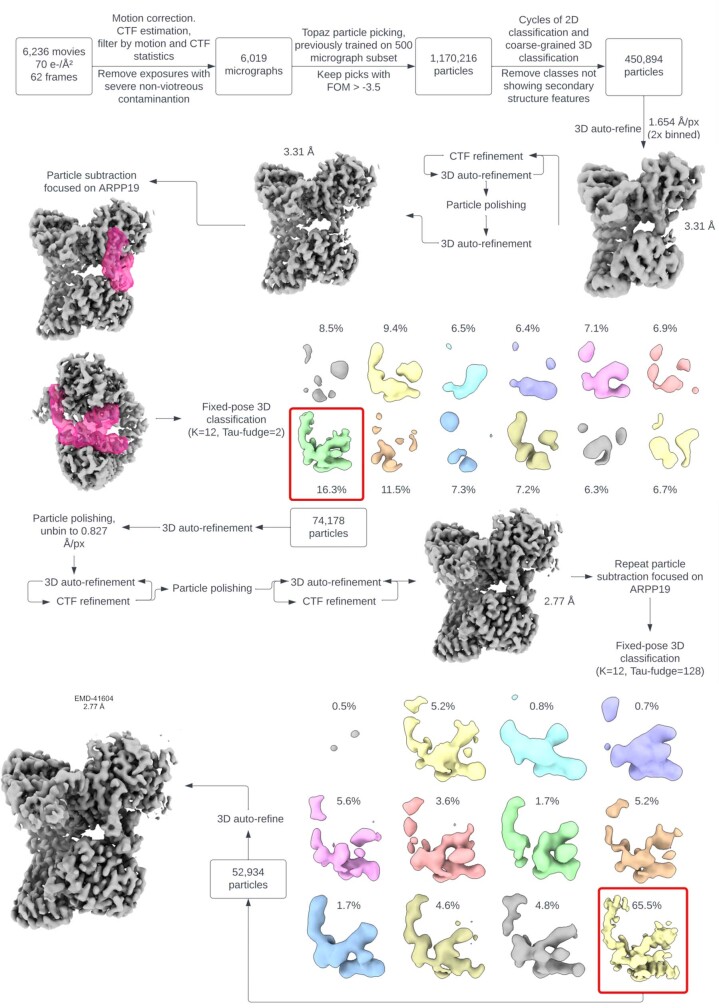
Cryo-EM image processing workflow for PP2A:B55-*tp*ARPP19. Particle counts and reconstruction resolutions are given at key junctions of the process. “Coarse grained” 3D classification denotes the inclusion of global angular and translational particle pose searches. All resolutions noted are calculated by the gold standard half-maps FSC = 0.143 criterion.

**Extended Data Fig. 7:**
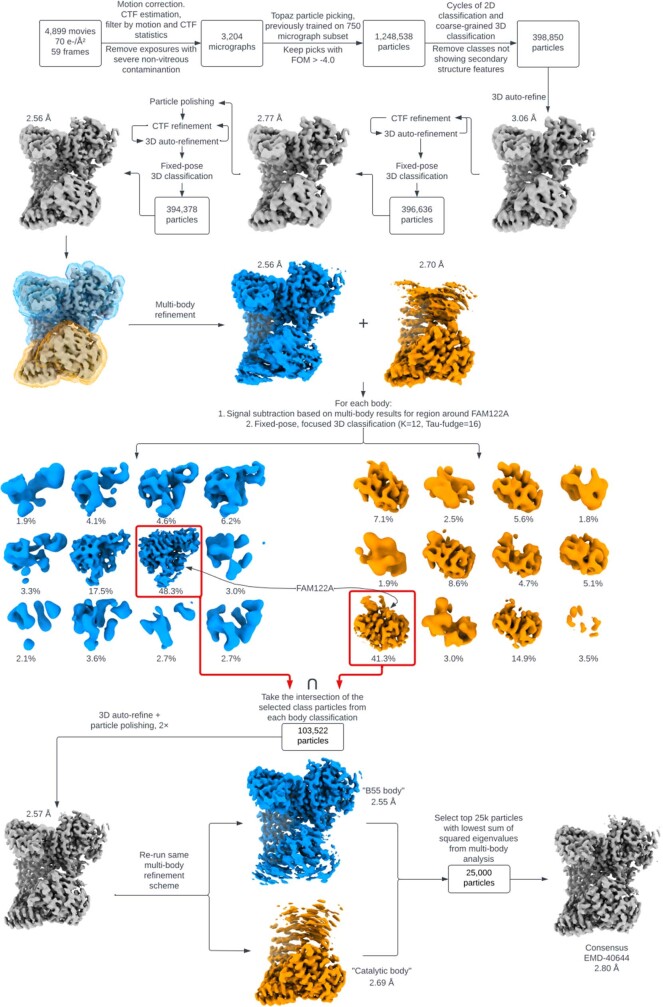
Cryo-EM image processing workflow for PP2A:B55-FAM122A. Particle counts and reconstruction resolutions are given at key junctions of the process. “Coarse grained” 3D classification denotes the inclusion of global angular and translational particle pose searches. All resolutions noted are calculated by the gold standard half-maps FSC = 0.143 criterion.

**Extended Data Fig. 8:**
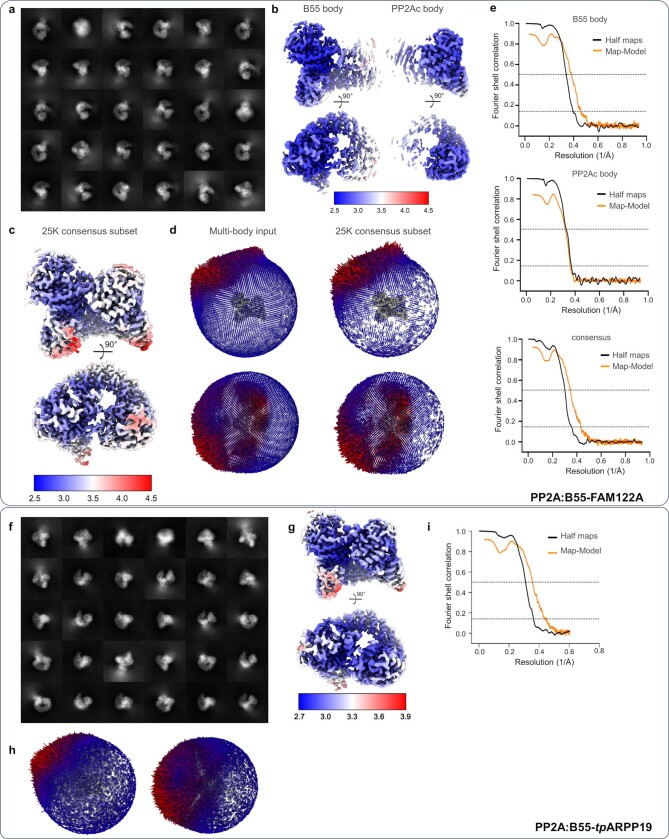
Cryo-EM 2D class averages and maps for PP2A:B55-inhibitor complexes. a-e, PP2A:B55-FAM122A; f-i, PP2A:B55-*tp*ARPP19. **a**. Reference-free 2D class averages generated from the 103,522 particles used in the final multi-body refinement. **b**. The multi-body refinement results for each body, B55 and PP2Ac, colored by local resolution. **c**. The 25,000-particle subset consensus map, colored by local resolution. **d**. Histograms of the particle pose angular distribution from the input to the final multi-body refinement (left) and the 25,000-particle consensus subset refinement (right). **e**. The Fourier shell correlation (FSC) for the independently refined half-maps (black) and the full map and atomic model (orange) for the multibody refinement results (B55 body, top, PP2Ac body, middle) and the 25,000-particle subset consensus (bottom). The “gold standard” half-maps FSC was calculated and corrected for masking effects using Relion; the map-model FSC was calculated by PHENIX using a mask around the model based on map resolution. The FSC = 0.5 and 0.143 thresholds are marked by dashed lines. For the B55 and PP2Ac multibody refinements, the half-maps FSC crosses the 0.143 threshold at 2.55 Å and 2.69 resolution, respectively, and the map-model FSC crosses the 0.5 threshold at 2.61 Å and 3.12 Å resolution, respectively. For the subset consensus refinement, the half-maps FSC crosses the 0.143 threshold at 2.8 Å resolution and the map-model FSC crosses the 0.5 threshold at 2.86 Å resolution. **f**. Reference-free 2D class averages generated from the 52,934 particles used in the final refinement. **g**. Cryo-EM map, colored by local resolution. **h**. Histograms of the particle pose angular distribution from the input to the final refinement. **i**. The Fourier shell correlation (FSC) for the refinement results. The “gold standard” half-maps FSC was calculated and corrected for masking effects using Relion; the map-model FSC was calculated by PHENIX using a mask around the model based on map resolution. The FSC = 0.5 and 0.143 thresholds are marked by dashed lines. For the refinement, the half-maps FSC crosses the 0.143 threshold at 2.77 Å resolution and the map-model FSC crosses the 0.5 threshold at 2.81 Å resolution.

**Extended Data Fig. 9:**
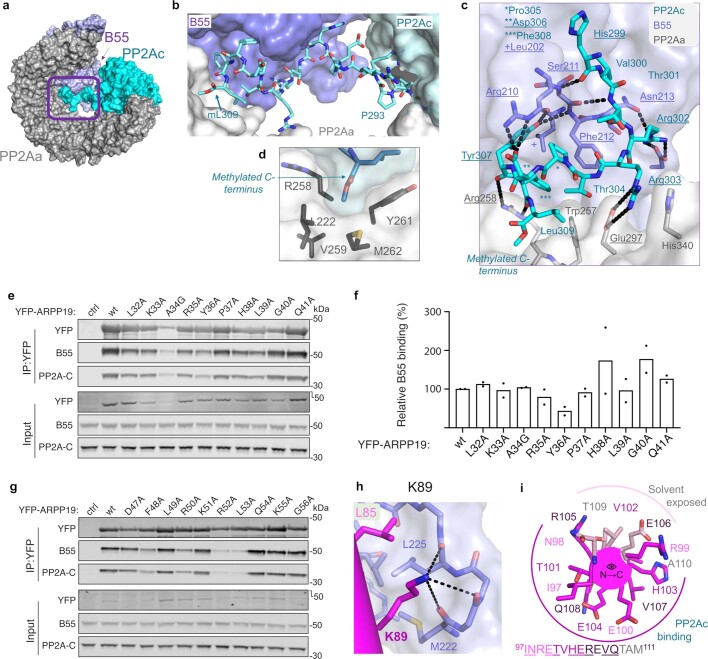
Intra- and intermolecular PP2A:B55-inhibitor interactions. **a**. The methylated C-terminal tail of PP2Ac (cyan) extends to the opposite side of PP2A (grey) where it interacts at the interface between PP2Aa and B55 (lavender). **b**. Close-up of (a) with the C-terminus shown as sticks and PP2Aa, B55 and the rest of PP2Ac shown as a surface. **c**. Different view of a,b with the PP2Ac C-terminus shown as sticks and PP2Aa and B55 interacting residues also shown as sticks. Polar/ionic inter-subunit interactions indicated by black dashed lines and the participating residues underlined. **d**. Detailed interactions between the methylated C-terminal tail of PP2Ac (cyan, highlighted by purple box in (a)) with PP2Aa (grey) and B55 (lavender). **e**. Alanine scanning mutagenesis of ARPP19 amino acids L32-Q41. The indicated YFP-ARPP19 constructs were transfected into HeLa cells and immunopurified. Binding efficiency of the YFP-ARPP19 derivatives to B55 and PP2Ac was determined by Western blotting. **f**. Quantification of (e) based on two independent experiments. **g**. Alanine scanning mutagenesis of ARPP19 amino acids D47-G56. The indicated YFP-ARPP19 constructs were transfected into HeLa cells and immunopurified. Binding efficiency of the YFP-ARPP19 derivatives to B55 and PP2Ac was determined by Western blotting. Quantification of (g) based on two independent experiments shown in Fig. 3e. **h**. FAM122A K89 makes polar/ionic interactions (dashes) with multiple residues from B55. **i**. Helical wheel N→C view of the B55 inhibition helix highlighting residues that interact with PP2Ac and those that are solvent exposed.

Following extensive sample optimization, we determined the structures of PP2A:B55–tpARPP19 and PP2A:B55–FAM122A_ID_ using cryo-EM at global resolutions of 2.77 and 2.80 Å, respectively (Fig. 2a,b and Extended Data Figs. 6–8). The previously solved PP2A:B55 crystal structure aided the modelling of the PP2Aa, B55 and PP2Ac subunits^29^. Compared with the PP2A:B55 crystal structure, the horseshoe-shaped conformation of PP2Aa contracted upon inhibitor binding (Fig. 2c). In both PP2A:B55–inhibitor maps, we observed continuous sections of density not accounted for by the PP2A:B55 crystal structure. The density common to both maps belongs to the PP2Ac C terminus (Fig. 2d, amino acids 294–309), which was not modelled in the PP2A:B55 crystal structure^29^. The C terminus extends across the PP2Aa central cavity to bind an extended pocket at the B55:PP2Aa interface, positioning mL309_C_ to bind a hydrophobic pocket in PP2Aa (Extended Data Fig. 9a–d; subscripts denote residues corresponding to the different subunits of the complexes as follows: A, PP2Aa; B, B55; C, PP2Ac; R, ARPP19; F, FAM122A). Overlaying the PP2A:B55–inhibitor complexes with PP2A:B56 (Protein Data Bank (PDB) ID: 2IAE^30^; superimposed using PP2Ac) showed that the PP2Ac mL309_C_ residues are more than 36 Å apart (Fig. 2e). The C-terminal interaction buries around 1,900 Å^2^ of solvent-accessible surface area, explaining the importance of this post-translational modification for PP2A:B55 complex formation and stability^19^.

The remaining unaccounted density corresponds to tpARPP19 or FAM122A (tpARPP19 residues 42–75 and 86–112; FAM122A residues 81–111). tpARPP19 binds exclusively to B55 and PP2Ac using helices connected by extended yet ordered loops (helices are pre-populated in free ARPP19; Extended Data Fig. 3a,c,i). This enables tpARPP19 to span the front surface of B55 and the PP2Ac active site. Furthermore, in a highly unusual conformation, tpARPP19 loops back on itself to form a stable, overlaid cross at the B55:PP2Ac interface. tpARPP19 binding buries more than 5,200 Å^2^ of solvent-accessible surface area. FAM122A also binds exclusively to B55 and PP2Ac and does so, again, using helices (pre-populated in free FAM122A; Extended Data Fig. 3d,f). FAM122A binds a short surface on B55 (the B55 binding platform) and across the PP2Ac active site; the interaction buries 2,700 Å^2^ solvent-accessible surface area. Both inhibitors bind the most conserved surface of B55 (Fig. 2f). Despite their common function, these structures show that tpARPP19 and FAM122A bind and inhibit PP2A:B55 using distinct mechanisms.

## B55-specific recruitment of tpARPP19

**Fig. 3:**
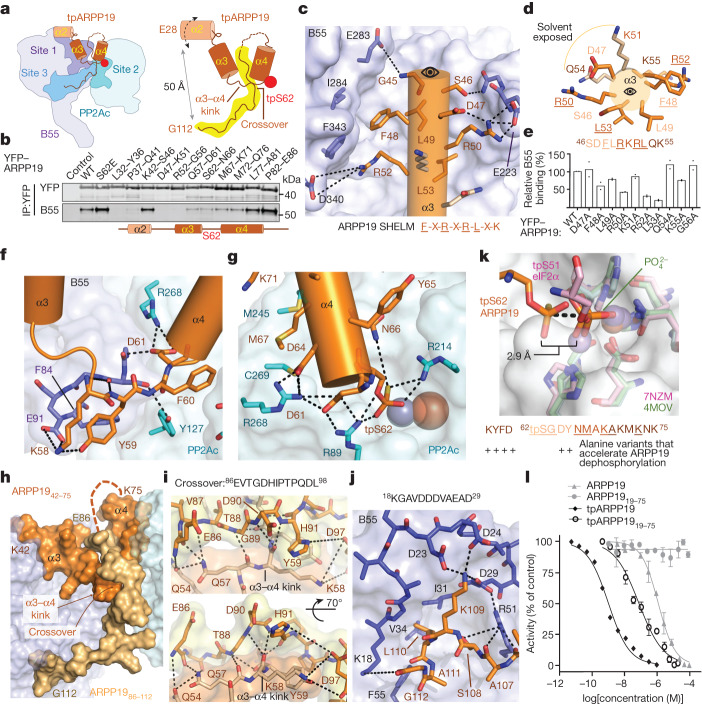
tpARPP19 inhibition of PP2A:B55. **a**, Cartoon of PP2A:B55–tpARPP19 with interaction sites labelled. **b**, 5-Ala mutational scan of ARPP19 L32–E86. YFP–ARPP19 variants were transfected into HeLa cells, immunopurified and B55 binding was determined by western blot (*n* = 2). ARPP19 α-helices shown as a cartoon. **c**, Interaction of helix α3 (orange) with B55 (lavender). ARPP19 binding SHELM shown below. **d**, Helix α3 helical wheel. B55-binding residues are in dark orange and solvent-exposed residues are in light orange. Label colours indicate residues in the same helical turn; B55-binding residues are underlined. **e**, Quantification of 1-Ala mutational scan of helix α3 (pull-down experiments performed as in **a**; *n* = 2). **f**, The ARPP19 α3–α4 kink. **g**, tpS62 at the PP2Ac active site. **h**, The ARPP19 crossover. Helices α3 and α4 are in orange; the remaining residues are in gold. **i**, The ARPP19 crossover. Intramolecular contacts are shown as dashed lines. **j**, ARPP19 binds B55 loop ^18^KGAVDDDVAEAD^29^. **k**, PPP active site overlay of PP2A:B55–tpARPP19. PP1 phosphate (PDB ID 4MOV; PP1, green) and a pre-dephosphorylation complex (PDB ID 7NZM; tpS51eIF2α, pink; PP1, light pink) are shown. tpARPP19 is in orange, PP2Ac is cyan. Bottom, residues that when mutated result in faster dephosphorylation of S62^15^. **l**, PP2A:B55 inhibition by ARPP19 variants (with or without phosphorylation; mean ± s.d.; *n* = 3 experimental replicates). Results representative of *n* = 3 independent experiments. Unpaired two-tailed *t*-test with 95% confidence interval was used to compare ARPP19 with tpARPP19_19–75_ (*P* < 0.0001) and tpARPP19 with tpARPP19_19–75_ (*P* = 0.0002). IC_50_ values are reported in Extended Data Table 1.

tpARPP19 binds PP2A:B55 using a tripartite mechanism: (1) residues 25–61 bind the top of B55 (B55 site 1); (2) tpS62 and helix α4 bind PP2Ac (B55 site 2); and (3) residues 86–112 bind B55 in a pocket (B55 site 3) nearly 50 Å away from site 1 (Fig. 3a). ARPP19 residues 25–41 include helix α2 (25–34) and bind the cleft between B55 loops L4/5 (L4/5 refers to the loop connecting β-propellers 4 and 5) and between L5/6 (B55 adopts a WD40 fold composed of 7 β-propellers connected by 7 loops). The density for these residues is present but amorphous, suggesting that α2 remains somewhat mobile in the B55 bound state (a fuzzy interaction^24,31,32^). The NMR data show that these residues interact with B55, as the intensities of these N/H^N^ cross-peaks are reduced when bound to either PP2A:B55 or B55_LL._ To test α2 binding to PP2A:B55 in cells, we generated YFP–ARPP19 variants in which either five amino acids (5-Ala, ^32^AAAAA^36^ or ^37^AAAAA^41^) or one amino acid (32–41) were changed to alanine (or A34G) and then tested their ability to pull down PP2A:B55 from cells (Fig. 3b and Extended Data Fig. 9e,f). Although only a single point variant exhibited reduced B55 and PP2Ac binding (Y36A), both 5-Ala variants of YFP–ARPP19—32–36 and 37–41—were unable to pull down B55. These data show that ARPP19 residues 25–41 contribute to B55 binding.

Following helix α2, the density for tpARPP19 is well defined, extending down towards the B55 platform, defined by β-propellers 2–4 and loops L1/2, L2/3 and L3/4. These interactions position ARPP19 helix α3 (^46^SDFLRKRLQK^55^) to bind the B55 platform with all ARPP19 residues except K51_R_ and Q54_R_ making multiple interactions with B55 (Fig. 3c,d). Because these residues form a helix, amino acids separated in sequence are adjacent in space. F48_R_ and R52_R_ form a π-stack and hydrophobic contacts with B55 L4/5 and L5/6 (I284_B_/Y337_B_/F343_B_), whereas L49_R_ and L53_R_ form hydrophobic contacts with L2/3 and L3/4 (Y178_B_/L198_B_/L225_B_/V228_B_). R50_R_ and R52_R_ also stabilize L3/4 or L5/6, respectively, via a salt bridge (E223_B_/D340_B_) (Fig. 3c). The key interactions between B55 and ARPP19 are mediated by ARPP19 residues F-L-R-X-R-L-X-K. Because these residues in ARPP19 are helical, and not extended, we refer to this sequence as a short helical motif (SHELM). To test whether these ARPP19 residues contribute to B55 binding in cells, we generated 5-Ala and single point variants for the ARPP19 α2–α3 loop and helix α3 and tested their ability to pull down PP2A:B55 from cells. Although B55 binding to the ^42^AAAAA^46^ variant was unchanged, the ^47^AAAAA^51^ and ^52^AAAAA^56^ variants could not pull down B55 (Fig. 3b). Multiple single alanine mutations for ARPP19 residues 47–56 also exhibited reductions in B55 and PP2Ac binding, particularly L53A and R52A (less than 25% compared with wild type), R50A and F48A (around 50% compared with wild type) and L49A and K55A (approximately 75% compared with wild type) (Fig. 3e and Extended Data Fig. 9g). These data are fully consistent with the structure, as mutating residues that interact with B55 (F48, L49, R50, R52, L53 and K55) reduce binding, whereas those that are mostly solvent-accessible (D47, K51, Q54 and G56) do not.

Next, ARPP19 extends towards the B55 L1/2 loop, where it kinks by 180° to bind to PP2Ac (Fig. 3a,f). Here, Y59_R_, F60_R_ and D61_R_ are splayed apart, with Y59_R_ binding B55, F60_R_ binding PP2Ac and D61_R_ binding both B55 and PP2Ac. These interactions position tpS62 directly above the metal ions in the PP2Ac active site, where it forms bipartite salt bridges with the substrate-coordinating arginine residues, R89_C_ and R214_C_ (Fig. 3g). This interaction is further stabilized by D61_R_ and D64_R_, which form salt bridges with R268_C_ and R89_C_, resulting in an extended network of ionic interactions between ARPP19 and PP2Ac that stabilize the tpS62 conformation. ARPP19 helix α4 (^62^tpSGDYNMAKAKMKNK^75^) extends from the PP2Ac active site towards the PP2Ac C terminus. In this way, ARPP19 helices α3 and α4 interact with B55 (α3) and PP2Ac (α4) using a helix-turn-helix ‘V’ conformation. ARPP19 residues ^76^QLPTAAPD^83^ remain mobile when bound to PP2A:B55. Consistent with the structure, pull-down experiments using YFP–ARPP19 5-Ala variants, in which the kink and helix α4 residues are mutated to alanine (^57^AAAAA^61^, ^62^AAAAA^66^, ^67^AAAAA^71^ and ^72^AAAAA^76^), weaken B55 binding, albeit not to the same extent as 5-Ala variants of α2 or α3 (Fig. 3b).

## The ARPP19 crossover

In a highly unusual conformation, the ARPP19 residues following the mobile 76–85 loop back towards the PP2Ac active site. This positions tpARPP19 residues ^86^EVTGDHIPTPQDL^98^ to cross over and stabilize the splayed Y59_R_ and F60_R_ residues at the B55:PP2Ac interface (referred to as the crossover; Fig. 3a,h,i). This interaction leverages hydrophobic and polar contacts, with E86_R_/V87_R_/T88_R_ binding tpARPP19 (F60_R_/Y65_R_/A68_R_) and I92_R_/P93_R_/P95_R_/L98_R_ binding tpARPP19, PP2Ac and B55 (Y59_R_/F60_R_, V126_C_/Y127_C_/G215_C_ and F84_B_/S89_B_). G89_R_, which lacks a Cβ atom, facilitates the close approach needed for backbone hydrogen bonding between the ARPP19 residues ^57^QKYF^60^ and ^89^GDH^91^. Together, these intra- (tpARPP19) and inter- (tpATPP19, PP2Ac and B55) molecular interactions stabilize the inhibitory conformation of ARPP19 (Fig. 3i). After the crossover, ARPP19 residues ^100^QRKPAL^105^ (S104A prevents phosphorylation of S104 by MASTL; Extended Data Fig. 3g) make limited contact with B55. Despite this, they are ordered because the remaining residues—^106^VASKLAG^112^, especially L110_R_—bind a hydrophobic pocket between B55 β-propellers 1 and 2, enabling K109_R_ to interact with multiple acidic residues in B55 loop L7/1 (^22^DDDVAEAD^29^) (Fig. 3j). The interaction of the ARPP19 C terminus with B55, independent of phosphorylation state, is fully consistent with our NMR data (Fig. 1d,g and Extended Data Fig. 4a–d).

## ARPP19-mediated inhibition of PP2A:B55

MASTL-phosphorylated ARPP19 is both an inhibitor and substrate of PP2A:B55^4–6,15,18^. To understand why MASTL-phosphorylated ARPP19 is only slowly dephosphorylated by PP2A:B55, we overlaid the structures of PP2A:B55–tpARPP19 via the PP2Ac subunit with the PPP subunit of both a PPP product complex (PP1 with a phosphate bound at the active site, PDB ID: 4MOV^26^) and a PPP pre-dephosphorylation complex (phosphorylated eIF2α trapped by the catalytically deficient PP1 D64A variant, PDB ID: 7NZM^33^). The thiophosphate and phosphate in the pre-dephosphorylation and product complexes overlap nearly perfectly. By contrast, the thiophosphoryl group of tpARPP19 is approximately 3 Å further away from both metal ions, in a position that is unproductive for dephosphorylation (Fig. 3k). This inhibitory conformation is stabilized by the interactions between the ARPP19 MASTL recognition residues (^58^KYFDSGDY^65^) with B55 and PP2Ac and the ARPP19 crossover (^86^EVTGDHIPTPQDL^98^), which is secured in place by the interaction of the ARPP19 C terminus at B55 site 3. Consistent with this, previous data showed that mutating either the MASTL recognition residues (K58A, Y59A, F60A, D61A, D64A and Y65A) or deleting the C terminus converts ARPP19 into a substrate^15^ (resulting in faster dephosphorylation; Fig. 3k). Similarly, comparing the half-maximal inhibitory concentrations (IC_50_) of ARPP19 and a C-terminal deletion (ARPP_19–75_) with and without thiophosphorylation shows that the interaction of the C terminus with B55 is essential for the potent inhibition of PP2A:B55 (Fig. 3l and Extended Data Table 1), as the C-terminal deletion variants either do not inhibit (non-phosphorylated ARPP19 versus ARPP19_19–75_) or become a more than 50-fold weaker inhibitor (tpARPP19 versus tpARPP19_19–75_). These overlapped structures also suggest that the mechanism by which ARPP19 becomes a substrate involves a shift of pS62 in the active site to a position in which the metal ion-activated nucleophilic water can mediate dephosphorylation. Our data suggest this is most probably achieved by the release of the ARPP19 C-terminal tail from B55.

## Inhibition of PP2A:B55 by FAM122A

**Fig. 4:**
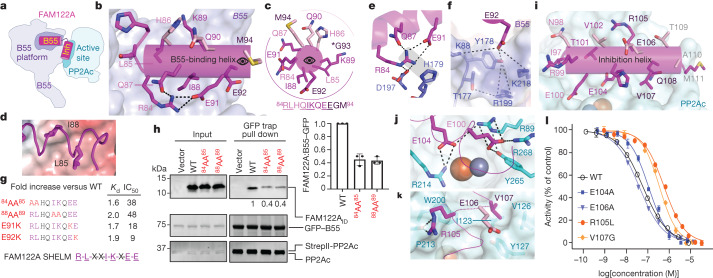
FAM122A inhibition of PP2A:B55. **a**, Cartoon of PP2A:B55–FAM122A. B55, B55-binding helix; inh, inhibition helix. **b**, FAM122A residues R84–E92 (magenta) bind B55 (lavender) via a three-turn helix. **c**, Helical wheel of the B55-binding helix. B55-binding residues are in magenta, solvent-exposed residues are light pink, and label colours indicate residues within the same helical turn. **d**, L85–I88 binding pocket on B55 (shown as an electrostatic potential surface). **e**, Intra- and intermolecular interactions of Arg84 with B55 (polar or ionic interactions are shown as dashes). **f**, Intermolecular interactions of E92 with B55 (polar or ionic interactions are shown as dashes). **g**, Mutations in the FAM122A SHELM negatively affect PP2A:B55 binding and inhibition (Extended Data Table 2). **h**, Pull-down assay with SHELM variants. expi293F lysates were co-transfected with GFP–B55 and PP2Ac was incubated with purified PP2Aa and FAM122A variants and immunopurified. FAM122A variant binding efficiency was determined by western blot. Quantification based on three independent experiments (mean ± s.d.). Unpaired two-tailed *t*-test with 95% confidence interval was used to compare wild-type FAM122A with SHELM variants (*P* < 0.0005). **i**, FAM122A residues I97–M111 (magenta) bind PP2Ac (cyan). **j**, Intermolecular interactions of E100 and E104 with PP2Ac (ionic interactions are shown as dashed lines). **k**, Intermolecular interactions of R105 and V107 with PP2Ac. **l**, PP2A:B55 inhibition by FAM122A_ID_ variants (mean ± s.d.; *n* = 3 experimental replicates). Results representative of *n* = 3 independent experiments. Unpaired two-tailed *t*-test with 95% confidence interval was used to compare wild-type FAM122A with E104A (*P* < 0.0001), E106A (*P* = 0.013), R105L (*P* < 0.0001) or V107G (*P* < 0.0001). IC_50_ value reported in Extended Data Table 1.

The interaction of FAM122A with PP2A:B55 is different to that of ARPP19, with FAM122A residues 81–111 binding PP2A:B55 with two helices (Fig. 4a). Helix α1 (the B55-binding helix) binds B55 and helix α2 (the inhibition helix) binds PP2Ac and blocks the active site. Although FAM122A residues 29–66 were not sufficiently ordered to be modelled, our NMR and binding data suggest that they contribute to binding via a dynamic (fuzzy) charge–charge interaction^24,31,32^ (Extended Data Table 2 and Extended Data Fig. 2b). The FAM122A B55-binding helix binds the B55 platform, with its N terminus pointing towards the centre of B55 and its C terminus pointing towards PP2Ac (Fig. 4b,c). Residues L85_F_ and I88_F_ are adjacent in space, which enables them to bind the same hydrophobic pockets on B55 used by ARPP19 (L49_R_/L53_R_) (Fig. 4d). Residue R84_F_ forms intramolecular polar and ionic interactions with Q87_F_ and E91_F_ that stabilize the helix (Fig. 4e). These interactions also allow R84_F_ to form a bidentate salt bridge with D197_B._ Lys89_F_ binds the carbonyls of L3/4 residues M222_B_, E223_B_ and L225_B_ (Extended Data Fig. 9h). Finally, E92_F_ binds a deep, basic pocket below the B55 platform where it coordinates residues from L1/2, L2/3 and L3/4 (Fig. 4f). The key interactions between B55 and FAM122A are mediated by FAM122A residues R-L-X-X-I-K-X-E-E, four of which (R84_F_/K89_F_/E91_F_/E92_F_) are highly conserved (Extended Data Fig. 1a). Mutating the basic–hydrophobic residue pairs—that is, ^84^RLHQIKQEE^92^ to ^84^AAHQIKQEE^92^ (^84^AA^85^) and ^84^RLHQAAQEE^92^ (^88^AA^89^)—reduced FAM122A binding by 1.6- and 2.0-fold, respectively (Fig. 4g and Extended Data Table 2). Pull-down assays using PP2A:B55 lysates incubated with ^84^AA^85^ and ^88^AA^89^ FAM122A showed similar reductions in binding compared with the wild-type protein (Fig. 4h). Consistent with their weaker affinities, the IC_50_ values of the ^84^AA^85^ and ^88^AA^89^ variants increased by 38- and 48-fold, respectively (Fig. 4g and Extended Data Table 1). Because the FAM122A E92K mutation was identified in cancer tissues (cBioPortal), we also generated E91K and E92K variants and showed they also bound PP2A:B55 less strongly and were less potent inhibitors of PP2A:B55 (Extended Data Tables 1 and 2 and Extended Data Fig. 2a,b).

Like ARPP19, FAM122A also binds PP2Ac (Fig. 4a,i). FAM122A residues C-terminal to the B55 helix form a sharp turn with helix α2 (^97^INRETVHEREVQTAM^111^, the inhibition helix; Extended Data Fig. 9i) binding and blocking the PP2Ac active site. This interaction is stabilized by hydrophobic and electrostatic interactions. L96_F_ and I97_F_ bind a hydrophobic pocket, positioning E100_F_ and E104_F_ to bind substrate-coordinating residues R268_C_/R89_C_ and R214_C_/R89_C_, respectively (Fig. 4j). The E104A variant only modestly weakens FAM122A inhibition (twofold; as a control, the inhibitory capacity of E106A, a solvent-accessible residue, was not affected; Extended Data Table 1). This suggests that interactions at the active site are not essential for PP2Ac inhibition, but instead may be due to interactions, such as those of R105_F_ and V107_F_, that stabilize the helix across the active site (Fig. 4k). cBioPortal^34^ highlighted that FAM122A R105L, V107G variants are present in different cancers (FAM122A is a tumour suppressor, as patients with cancer who express low levels of FAM122A have significantly worse overall survival than those with high levels of expression^8^). FAM122A R105L and V107G variants showed 11- and 6-fold less inhibition than the wild-type protein, respectively (Fig. 4l and Extended Data Table 1), demonstrating that the probable mode of action of these cancer variants is due to a weaker inhibition of PP2A:B55, thereby disrupting PP2A:B55 cellular functions.

## Substrate recruitment via B55

**Fig. 5:**
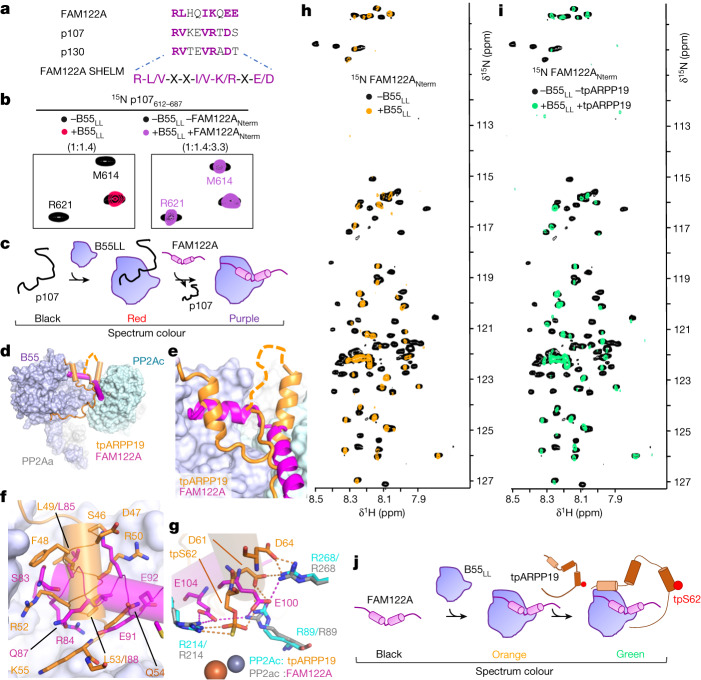
PP2A:B55 recruitment. **a**, PP2A:B55 SHELM sequences from experimentally confirmed B55 interactors. **b**, Left, 2D ^1^H,^15^N HSQC spectrum of ^15^N-labelled p107 alone and with B55_LL_ (p107:B55 ratio is shown). Right, 2D ^1^H,^15^N HSQC spectrum of ^15^N-labelled p107 alone and with B55_LL_ and an excess of unlabelled FAM122A (p107:B55:FAM122A_Nterm_ ratio is shown). **c**, Model of FAM122A-mediated displacement of p107. **d**, Overlay of PP2A:B55 bound to tpARPP19 or FAM122A. **e**, Magnified view of the overlapping regions shown in **d**. **f**, Overlay of tpARPP19 (orange) or FAM122A (magenta) at the B55 platform. **g**, Overlay of tpARPP19 (orange) or FAM122A (magenta) at the PP2Ac catalytic pocket. PP2Ac residues from the ARPP19 complex are labelled in cyan and those from the FAM122A complex are labelled in grey. Ionic interactions are shown as dashed lines. Metal ions in PP2Ac are shown as spheres. **h**, 2D ^1^H,^15^N HSQC spectrum of ^15^N-labelled FAM122A with and without B55_LL_. **i**, 2D ^1^H,^15^N HSQC spectrum of ^15^N-labelled FAM122A with and without B55_LL_ and unlabelled tpARPP19. **j**, Model of FAM122A and ARPP19 binding B55_LL_ simultaneously.

**Extended Data Fig. 10:**
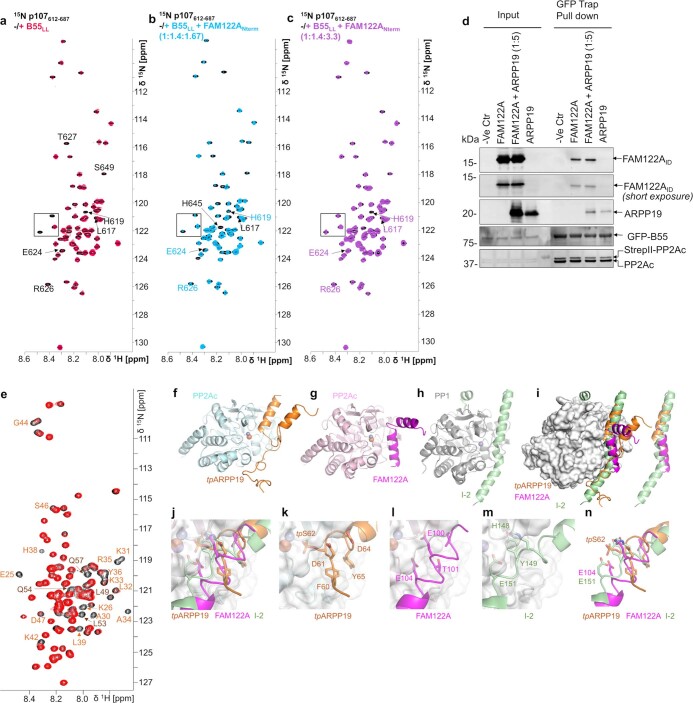
NMR displacement and pull-down studies show that FAM122A displaces p107 and that FAM122A and *tp*ARPP19 can bind simultaneously, with similarities to PP1. **a**. 2D [^1^H,^15^N] HSQC spectrum of ^15^N-labeled p107 alone (black) and in complex with B55_LL_ (red) shows that specific p107 residues bind B55_LL_. **b**. The addition of unlabeled FAM122A replaces ^15^N-labeled p107 in the identical B55_LL_ binding surface and now starts to appear in the 2D [^1^H,^15^N] HSQC spectrum (light blue) in an identical position as in the 2D [^1^H,^15^N] HSQC spectrum of ^15^N-labeled p107 alone (black). **c**. Further addition of unlabeled FAM122A replaces ^15^N-labeled p107 in the identical B55_LL_ binding surface and now nearly fully appears in the 2D [^1^H,^15^N] HSQC spectrum (violet) in an identical position as in the 2D [^1^H,^15^N] HSQC spectrum of ^15^N-labeled p107 alone (black). Black boxes highlight inserts shown in Fig. 5b in the manuscript. **d**. Pulldown assay demonstrating that *tp*S62-ARPP19 does not displace FAM122A when bound to PP2A:B55. Expi293F lysates co-transfected with GFP-B55 and PP2Ac-strep were incubated with purified PP2Aa and FAM122A alone and with a 5-fold surplus of *tp*S62-ARPP19, pulled-down using a GFP-TRAP and immunoblotted for the indicated proteins. Results representative of 3 independent experiments. **e**. 2D [^1^H,^15^N] HSQC spectrum of 4.5 μM ^15^N-labeled ARPP19 alone (black) and in complex with 6.9 μM unlabeled B55_LL_ and 22.5 μM unlabeled FAM122A (red) shows that mostly ARPP19 helix α2 H^N^/N cross peaks stay bound to B55_LL_ (annotated in orange); compare with Fig. 1f without FAM122A. **f**. *tp*ARPP19 (orange) bound to PP2Ac (cyan). **g**. FAM122A (magenta) bound to PP2Ac (light pink). **h**. I-2 (light green) bound to PP1 (grey; pdbid 2OG8). **i**. Overlay of PP2Ac and PP1 bound to *tp*ARPP19, FAM122A and I-2, respectively. Colors as in a-c. PP2Ac shown as surface. **j**. Zoom view of (i) with the residues near the active site shown as sticks. Bound metals are shown. **k**. PP2Ac:*tp*ARPP19 in same orientation as j. **l**. PP2Ac:FAM122A in same orientation as j. **m**. PP1:I-2 in same orientation as j. **n**. Same as j but without the PPPs.

PP2A:B55 dephosphorylates hundreds of substrates^35,36^, including p107 and p130, whose binding domains share sequence similarity with the B55 helix of FAM122A^37^ (Fig. 5a). To test whether their B55 binding sites overlap, we performed an NMR competition assay (Fig. 5b). First, we formed a complex between ^15^N-labelled p107 and B55_LL_ and identified all p107 N/H^N^ cross-peaks that lost intensity due to B55_LL_ binding. We then added an excess of unlabelled FAM122A_Nterm_ and monitored for p107 displacement from B55. All p107 residues that had reduced N/H^N^ cross-peaks intensities due to B55 binding regained their intensities in the presence of excess FAM122A, showing that FAM122A displaced p107 from B55 (Fig. 5b and Extended Data Fig. 10a–c). These results establish that, in addition to ARPP19 and FAM122A, p107 (and probably other substrates) uses the B55 platform to bind B55, demonstrating that ARPP19 and FAM122A, in addition to inhibiting the active site, also block substrate binding to PP2A:B55 (Fig. 5c).

## Simultaneous binding of both inhibitors

ARPP19 and FAM122A share two PP2A:B55 interaction sites: the B55 platform and the PP2Ac active site (Fig. 5d,e). However, their detailed interactions differ. Overlaying both complexes via B55 shows that L53_R_ and I88_F_ bind the B55 platform central hydrophobic pocket, whereas L49_R_ and L85_F_ bind an adjacent, shallower hydrophobic pocket (Fig. 5f). The number of intervening residues between these two corresponding hydrophobic amino acids are not identical (ARPP19 has four, whereas FAM122A has three) as the orientations of the bound helices differ. Similarly, whereas R52_R_ forms a π-stacking interaction and salt bridge with D340_B_, R84_F_ binds in a pocket nearly 10 Å away to form a salt bridge with D197_B_. These differences are again due to the distinct binding orientations of these helices. The second shared binding site is the PP2Ac active site (Fig. 5g). Although both inhibitors use helices to bind PP2Ac, these helices project in opposite directions, with ARPP19 helix α4 extending towards the PP2Ac C terminus whereas the FAM122A inhibitory helix extends towards the PP2Ac hydrophobic groove. The only area of overlap is at the active site itself, where tpS62_R_ projects deeply into the active site, whereas E100_F_ or E104_F_ bind at the periphery.

The remainder of the interactions are unique, with ARPP19 binding B55 at additional interaction sites via helix α2 and its C terminus. This suggests that ARPP19 and FAM122A, which have similar affinities for PP2A:B55 (Extended Data Table 2) may bind PP2A:B55 simultaneously. To test this, we used NMR and pull-down assays. We first formed the complex between B55_LL_ and ^15^N-labelled FAM122A_Nterm._ (Fig. 5h) and then added tpARPP19 (Fig. 5i). Despite an excess of around 2.5-fold of tpARPP19, no change in the 2D ^1^H,^15^N HSQC spectrum of the FAM122A_Nterm_ was observed, demonstrating that FAM122A_Nterm_ was not displaced by tpARPP19 (Fig. 5i). We also performed a pull-down competition assay by affinity purification of PP2A:B55 (using GFP–B55) in the presence of FAM122A alone or FAM122A with a fivefold excess of tpARPP19 (Extended Data Fig. 10d). Not only were both FAM122A and tpARPP19 pulled down with PP2A:B55, but the amounts of FAM122A pulled down in the absence or presence of tpARPP19 were identical. Finally, we performed the reverse NMR experiment (B55_LL_ bound to ^15^N-labelled ARPP19 and then adding excess unlabelled FAM122A_Nterm_), which showed that ARPP19 stays bound to B55, predominantly via helix α2, in the presence of FAM122A (Extended Data Fig. 10e). Together, these experiments show that FAM122A_Nterm_ and tpARPP19 can bind PP2A:B55 simultaneously (Fig. 5j), and thus, that B55 uses its multiple, distinct interaction surfaces to differentially engage B55-specific regulators and/or substrates.

## Discussion

The inhibition of PP2A:B55 by two B55-specific inhibitors, FAM122A and ARPP19, is essential for mitotic entry^7,8,38,39^. Our data reveal their unexpected modes of PP2A:B55 binding and inhibition, providing a detailed understanding of their function. These data show that PP2A:B55 binds its regulators in a different manner to other PPPs. PP1^24,27,40^, PP2A:B56^28,41^, calcineurin^25,42^ (PP2B–PP3) and PP4^43^ recruit their cognate regulators and substrates using PPP-specific SLIMs^44^. By contrast, PP2A:B55 recruits its regulators ARPP19 and FAM122A using α-helices. Different to PPP–SLIM interactions, which are anchored by hydrophobic residues that bind to deep hydrophobic pockets, the B55 platform is comparatively flat with shallow hydrophobic pockets. The lack of pocket depth allows hydrophobic residues to approach and bind via multiple orientations (Fig. 5f), rather than the single orientation observed in SLIM interactions^45^. In addition, the B55 platform is bordered by charged residues, especially acidic residues. Because basic residues (arginine and lysine) are long, when present in B55 binding helices, they form salt bridges using an array of conformations, as necessitated by the helical binding orientation (Figs. 3 and 4). Thus, and in contrast to PP2A:B56 or calcineurin, in which PPP-specific SLIM sequences have been used to identify putative substrates using sequence alone, our data suggests that the analogous strategy is not readily applicable for identifying novel PP2A:B55-specific substrates. For example, the sequences used by ARPP19 and FAM122A to bind the same pockets of the B55 platform are not highly conserved (for ARPP19, F-L-R-X-R-L-X-K; for FAM122A, R-L-X-X-I-K-X-E-E) and although some substrates, such as p107/p130, may share similarities with known binding sequences (that is, FAM122A), our data suggest that different B55 substrates may bind the platform via mechanisms not yet observed. Finally, B55 belongs to the WD40 propeller family, and thus adopts a fold that is an established protein interaction domain that has been demonstrated to bind other proteins using a diverse range of interactions^46^. These observations, coupled with the discovery that ARPP19 also binds B55 using its C terminus, suggests that B55 may recruit a set of substrates via interaction surfaces outside the B55 platform used by ARPP19, FAM122A and p107.

In addition to blocking substrate recruitment, our structures also show that FAM122A and tpARPP19 inhibit PP2A:B55 by blocking the PP2Ac active site (Figs. 3 and 4). This combined mechanism of inhibition (blocking substrate recruitment and inhibiting catalytic site access) is also used by other members of the PPP family, in particular PP1. Like FAM122A, protein phosphatase 1 inhibitor-2 (I-2) is an IDP inhibitor of PP1^47^ that both blocks PP1-specific substrate and regulator recruitment (by binding PP1-specific SLIM interaction sites, the SILK and the RVxF binding pockets) and blocks catalytic site access^47,48^ (by using a long helix to bind over the PP1 active site in a phosphorylation-independent manner) (Extended Data Fig. 10f–m). This shows that PPP family members PP1 and PP2A:B55 both have endogenous IDP inhibitors that use a common mechanism to potently inhibit their ability to dephosphorylate their cognate substrates, suggesting that this may be a mechanism present throughout the PPP family.

The current literature supports a model in which PP2A:B55 is initially inhibited by FAM122A and later by phosphorylated ARPP19^5,6,8,18,35^ (Fig. 1a), with the assumption that inhibitor binding is mutually exclusive. However, our NMR and pull-down data show that FAM122A and ARPP19 can bind PP2A:B55 simultaneously, with FAM122A binding the B55 platform, and ARPP19, leveraging its multiple B55 interaction sites, binding B55 predominantly via helix α2. The ability of two regulators that share a subset of interaction sites to bind simultaneously to their cognate PPP has been observed for other PPPs (that is, the PP1–spinophilin–I-2 complex^49,50^). In this case, spinophilin binds the PP1 RVxF SLIM interaction pocket, and the I-2 RVxF sequence releases from PP1; it is the extensive interactions of I-2 at the PP1 SILK binding pocket and active site that allows the I-2 RVxF sequence to be dispensable for PP1 binding. Here we show that ARPP19, FAM122A and PP2A:B55 form a similar complex, in which—in the presence of FAM122A—the interactions of ARPP19 at sites 2 and 3 are dispensable for binding. Whether and how these ternary interactions contribute to the regulation of PP2A:B55 activity during mitosis remain to be elucidated. These data also suggest that the stable dissociation of FAM122A from PP2A:B55 is needed for formation of the full inhibitory PP2A:B55–pARPP19 complex. One possibility is that a currently unidentified post-translational modification dissociates FAM122A from PP2A:B55 (such as phosphorylation; phosphorylation of FAM122A S37 has already been shown to quantitatively dissociate FAM122A from PP2A:B55 to activate the G2/M checkpoint^8^). This would enable the formation of the full PP2A:B55–pARPP19 inhibitory complex and, once formed, serve as a ‘timer’ to facilitate mitotic exit via the slow transition of pARPP19 from an inhibitor to a substrate. The molecular bases for these events are under active investigation. Together, these studies provide a molecular understanding of regulator and substrate recruitment of the PP2A:B55 holoenzyme. Because of the key regulatory functions of PP2A:B55 in mitosis and DNA damage repair, these data provide a roadmap for characterizing disease-associated mutations and pursuing new avenues to therapeutically target this complex, by individually blocking a subset of regulators that use different B55 interaction sites.

## Methods

### Bacterial protein expression

PP2Aa_9–589,_ FAM122A_1–124_ (FAM122A_Nterm_), FAM122A_29–120_ (FAM122A_ID_), FAM122A_67–120_, ARPP19, ARPP19_S104A_, ARPP19_19–75_ and p107_612–687_ were subcloned into pTHMT containing an N-terminal His_6_-tag followed by maltose binding protein (MBP) and a tobacco etch virus (TEV) protease cleavage site. For expression, plasmid DNAs were transformed into *Escherichia coli* BL21 (DE3) RIL or BL21 (DE3) cells (Agilent). Freshly transformed cells were grown at 37 °C in LB broth containing kanamycin antibiotics (50 µg ml^−1^) until they reached an optical density (OD_600_) of ~0.8. Protein expression was induced by addition of 1 mM β-d-thiogalactopyranoside (IPTG) to the culture medium, and cultures were allowed to grow overnight (18–20 h, 250 rpm shaking) at 18 °C. Cells were collected by centrifugation (8,000*g*, 15 min, 4 °C) and stored at −80 °C until purification. Expression of uniformly ^13^C- and/or ^15^N-labelled protein was carried out by growing freshly transformed cells in M9 minimal medium containing 4 g l^−1^ [^13^C]-d-glucose and/or 1 g l^−1^ ^15^NH_4_Cl (Cambridge Isotopes Laboratories) as the sole carbon and nitrogen sources, respectively. FAM122A_ID_ variants E92K, R105L, V107G, S120C, E104A/S120C, E106A/S120C, R84A/L85A/S120C, I88A/K89A/S120C, E91K/S120C, E92K/S120C, FAM_67-120_S120C and ARPP19/S10C were generated by site-directed mutagenesis, sequence verified and expressed as described above.

### Cell culture

Expi293F cells were obtained from ThermoFisher (A14527) and grown in HEK293 Cell Complete Medium (SMM293-TII, Sino Biological M293TII). For transient overexpression of B55 and PP2Ac constructs, cells were transfected using polyethyleneimine (PEI) transfection reagent. For western blot and immunoprecipitation studies, whole-cell extracts were prepared by lysing cells in ice-cold lysis buffer (20 mM Tris pH 8.0, 500 mM NaCl, 0.5 mM TCEP, 1 mM MnCl_2_, 0.1% Triton X-100, Phosphatase inhibitor cocktail (ThermoFisher)), sonicating and clearing the lysate by centrifuging at 15,000*g* for 20 min at 4 °C. Total protein concentrations were measured using the Pierce 660 Protein Assay Reagent (ThermoFisher).

### Mammalian protein expression

Full-length B55_1–477_ was cloned into pcDNA3.4 including an N-terminal green fluorescence protein (GFP) followed by a TEV cleavage sequence. Full-length PP2Ac_1–309_ was cloned into pcDNA3.4 with an N-terminal Strep tag followed by a TEV cleavage sequence. B55 loopless (B55_LL_), in which B55 residues 126–164 that interact directly with PP2Aa were removed and replaced with a single NG linker (Fig. 1b), was cloned into pcDNA3.4 with an N-terminal GFP followed by a TEV cleavage sequence. All plasmids were amplified and purified using the NucleoBond Xtra Maxi Plus EF (Macherey-Nagel). B55_WT_ and B55_LL_ were individually expressed in Expi293F cells (ThermoFisher). B55_1–477_ and PP2Ac_1–309_ were co-expressed in Expi293F cells at a 1:2 DNA ratio.

Transfections were performed in 500 ml medium (SMM293-TII, Sino Biological) in 2 l flasks using polyethylenimine (Polysciences) reagent according to the manufacturer’s protocol in an incubator at 37 °C and 8% CO_2_ under shaking (125 rpm). On the day of transfection, the cell density was adjusted to 2.8 × 10^6^ cells per ml using fresh SMM293-TII expression medium. DNA of PP2Ac and B55 (2:1 ratio) were diluted in Opti-MEM Reduced Serum Medium (ThermoFisher). Similarly, in a separate tube, PEI (3× the amount of DNA) was diluted in the same volume of Opti-MEM Reduced Serum Medium (ThermoFisher). The DNA and PEI mixtures were combined and incubated for 10 min at room temperature, before being added to the cell culture. Valproic acid (2.2 mM final concentration, Sigma) was added to the cells 4 h after transfection and 24 h after transfection sterile-filtered glucose (4.5 ml per 500 ml cell culture, 45%, glucose stock) was added to the cell culture flasks to boost protein production. Cells were collected 48 h after transfection by centrifugation (2,000*g* for 20 min, 4 °C) and stored at −80 °C.

### FAM122A purification

Cell pellets expressing FAM122A_Nterm_, FAM122A_ID_ and FAM_67–120_ and variants were resuspended in ice-cold lysis buffer (50 mM Tris pH 8.0, 500 mM NaCl, 5 mM imidazole, 0.1% Triton X-100, EDTA-free protease inhibitor tablet (ThermoFisher)), lysed by high-pressure cell homogenization (Avestin Emulsiflex C3). Cell debris was pelleted by centrifugation (42,000*g*, 45 min, 4 °C), and the supernatant was filtered with 0.22-µm syringe filters (Millipore). The proteins were loaded onto a HisTrap HP column (Cytiva) pre-equilibrated with buffer A (50 mM Tris pH 8.0, 500 mM NaCl, 5 mM imidazole) and eluted using a linear gradient (0–60%) with buffer B (50 mM Tris pH 8.0, 500 mM NaCl and 500 mM imidazole). Fractions containing the protein were pooled and dialysed overnight at 4 °C with TEV protease (in house; His_6_ tagged) to cleave the His_6_–MBP tag. Following cleavage, the sample was either (1) loaded under gravity onto Ni^2+^-NTA beads (Prometheus) pre-equilibrated with buffer A, the flow through and wash A fractions were collected, and then twice heat purified (80 °C, 10 min) or (2) twice heat purified (80 °C, 10 min). Samples were centrifuged at 15,000*g* for 10 min to remove precipitated protein. Supernatant was concentrated and purified using size-exclusion chromatography (SEC; Superdex 75 26/60 (Cytiva)) in either NMR buffer (20 mM Na_2_HPO_4_/NaH_2_PO_4 _pH 6.3, 150 mM NaCl, 0.5 mM TCEP), IC_50_ assay buffer (20 mM Tris pH 8.0, 150 mM NaCl, 0.5 mM TCEP) or fluorescence polarization assay buffer (20 mM Tris pH 7.0, 150 mM NaCl, 0.5 mM TCEP). Samples were either directly used for NMR data collection or flash frozen and stored at −80 °C.

### ARPP19 purification

The protocol is identical for all ARPP19 constructs. Cell pellets were resuspended in lysis buffer (50 mM Tris-HCl pH 8.0, 500 mM NaCl, 5 mM imidazole, 0.1% Triton X-100, EDTA-free protease inhibitor (ThermoFisher)), lysed by high-pressure cell homogenization (Avestin C3-Emulsiflex), cell debris pelleted by centrifugation (42,000*g*, 45 min), and the supernatant was filtered with 0.22-µm syringe filters (Millipore). The proteins were loaded onto a HisTrap HP column (Cytiva) pre-equilibrated with buffer A (50 mM Tris pH 8.0, 500 mM NaCl, 5 mM imidazole), and eluted using a linear gradient (0–60%) of buffer B (50 mM Tris pH 8.0, 500 mM NaCl, 500 mM imidazole). Fractions containing the protein were pooled and dialysed overnight at 4 °C with TEV protease to cleave the MBP and His_6_ tags. The cleaved protein was incubated with Ni^2+^-NTA resin (Cytiva) and washed with buffer A. The flow through and wash A fractions were collected, and heat purified by incubating the samples at 80 °C for 20 min. The samples were centrifuged at 15,000*g* for 10 min to remove precipitated protein, concentrated and purified using SEC (Superdex 75 26/60 (Cytiva)) in NMR buffer (20 mM Na_2_HPO_4_/NaH_2_PO_4_ pH 6.3, 150 mM NaCl, 0.5 mM TCEP), IC_50_ assay buffer (20 mM Tris pH 8.0, 150 mM NaCl, 0.5 mM TCEP) or fluorescence polarization assay buffer (20 mM HEPES pH 7.0, 150 mM NaCl, 0.25 mM TCEP). Purified samples were again heat purified (80 °C for 5 min), centrifuged at 15,000*g* for 10 min to remove any precipitated protein, and were either directly used for NMR data collection or flash frozen and stored at −80 °C.

### PP2Aa purification

Cell pellets expressing PP2Aa_9-589_ were resuspended in ice-cold lysis buffer (50 mM Tris pH 8.0, 500 mM NaCl, 5 mM imidazole, 0.1% Triton X-100, EDTA-free protease inhibitor tablet (ThermoFisher)), lysed by high-pressure cell homogenization (Avestin Emulsiflex C3). Cell debris was pelleted by centrifugation (42,000*g*, 45 min, 4 °C), and the supernatant was filtered with 0.22-µm syringe filters. The proteins were loaded onto a HisTrap HP column (Cytiva) pre-equilibrated with buffer A (50 mM Tris pH 8.0, 500 mM NaCl, 5 mM imidazole) and eluted using a linear gradient (0 to 40%) with buffer B (50 mM Tris pH 8.0, 500 mM NaCl and 500 mM imidazole). Fractions containing the protein were pooled and dialysed overnight at 4 °C with TEV protease (in house; His_6_-tagged) to cleave the His_6_–MBP tag and loaded under gravity onto Ni^2+^-NTA beads (Prometheus) pre-equilibrated with buffer A. Flow through and wash A fractions were collected, concentrated and loaded onto QTrap HP column (Cytiva) for further purification. The proteins were eluted with a 100 mM–1 M salt gradient (buffer A: 20 mM Tris pH 8.0, 100 mM NaCl, 0.5 mM TCEP; buffer B: 20 mM Tris pH 8.0, 1 M NaCl, 0.5 mM TCEP). PP2Aa fractions were concentrated and further purified using SEC (Superdex 200 26/60 (Cytiva)) in assay buffer (20 mM Tris pH 8.0, 150 mM NaCl, 0.5 mM TCEP). Samples were either directly used or flash frozen and stored at −80 °C.

### MASTL expression and purification

Expi293F cells were transfected with pcDNA5_FRT_TO_3xFLAG_MASTL as described above. A cell pellet expressing MASTL was resuspended in ice-cold lysis buffer (20 mM Tris pH 8.0, 500 mM NaCl, 0.5 mM TCEP, 0.1% Triton X-100, EDTA-free protease inhibitor tablet (ThermoFisher)), lysed by high-pressure cell homogenization (Avestin Emulsiflex C3). Cell debris was pelleted by centrifugation (42,000*g*, 45 min, 4 °C), and the supernatant was filtered with 0.22-µm syringe filters (Millipore). Lysates were incubated with Anti-Flag M2 beads (Sigma), pre-equilibrated with wash buffer 1 (20 mM Tris pH 8.0, 500 mM NaCl and 0.5 mM TCEP) and slowly rocked at 4 °C for 2 h. Following, beads were washed 3 times with wash buffer (20 mM Tris pH 8.0, 500 mM NaCl, 0.5 mM TCEP, 1 mM MnCl_2_) and bound MASTL protein was eluted by incubating with 150 ng µl^−1^ 3× Flag peptide (Biosynthesis) for 10 min. Purified, active MASTL was mixed with 10% glycerol and stored at −80 °C.

### PKA expression and purification

For expression, PKA (human Cα1 in pet15b) was transformed into *E. coli* BL21 (DE3) RIL cells (Agilent). Freshly transformed cells were grown at 37 °C in LB broth until they reached an optical density (OD_600_) of ~0.8. Protein expression was induced by addition of 1 mM β-d-thiogalactopyranoside (IPTG) to the culture medium, and cultures were allowed to grow overnight (18–20 h, 250 rpm shaking) at 18 °C. Cells were collected by centrifugation (8,000*g*, 15 min, 4 °C) and stored at −80 °C until purification. For purification, cell pellets were resuspended in ice-cold lysis buffer (50 mM Tris pH 8.0, 500 mM NaCl, 5 mM imidazole, 0.1% Triton X-100, EDTA-free protease inhibitor (ThermoFisher)) and lysed by high-pressure cell homogenization (Avestin Emulsiflex C3). Cell debris was pelleted by centrifugation (42,000*g*, 45 min, 4 °C), and the supernatant was filtered with 0.22-µm syringe filters (Millipore). The proteins were loaded onto a HisTrap HP column (Cytiva) pre-equilibrated with buffer A (50 mM Tris pH 8.0, 500 mM NaCl, 5 mM imidazole) and eluted using a linear gradient (0–80%) with buffer B (50 mM Tris pH 8.0, 500 mM NaCl, 500 mM imidazole). Fractions containing the protein were pooled and dialysed overnight in the buffer (20 mM Tris pH 8, 50 mM NaCl, 1 mM EDTA, 2 mM DTT) at 4 °C. Purified sample was centrifuged at 15,000*g* for 10 min to remove precipitated protein. Supernatant protein sample was mixed with 50% glycerol and stored at −80 °C.

### Phosphorylation of ARPP19

Purified ^15^N-labelled-ARPP19 (25 μM) was incubated with either PKA or MASTL kinase (10:1 ratio) in phosphorylation buffer (100 mM Tris pH 7.5, 2 mM DTT, 10 mM MgCl_2_) with 500 µM of ATP-γ-S or ATP (Sigma) for thiophosphorylation and phosphorylation. The kinase reaction was left at 37 °C for 72−90 h. Phosphorylated ARPP19 was heat purified by incubating the samples at 80 °C for 10 min. The samples were centrifuged at 15,000*g* for 10 min to remove precipitated kinase and either immediately used for experiments or flash frozen and stored at −80 °C. Complete phosphorylation was confirmed by chemical shift changes of the phosphorylated serine residue(s) using 2D ^1^H,^15^N HSQC spectra.

### Immunoprecipitation and western blot for B55 versus B55_LL_ interaction with PP2Aa

GFP-tagged B55 or B55_LL_ and associated endogenous proteins were captured by incubating equal amounts of total protein (~500 µg) for each condition with GFP-Trap nanobody agarose beads (prepared using AminoLink Plus Immobilization Kit; ThermoFisher) at 4 °C for 16 h. Following 3 washes with wash buffer (20 mM Tris pH 8.0, 500 mM NaCl, 0.5 mM TCEP, 1 mM MnCl_2_), bound proteins were eluted with 2% SDS sample buffer (90 °C, 10 min), resolved by SDS–PAGE (Bio-Rad) and transferred to PVDF membrane for western blot analysis using indicated antibodies (see Reporting summary). Purified PP2A:B55 complex was used as a positive control. Antibody fluorescence signals were captured using a ChemiDoc MP Imaging System (Image Lab Touch Software 2.4; Bio-Rad) and band intensities quantified using ImageJ 1.53t^51,52^.

### FAM122A interaction with PP2A:B55 complex

Purified FAM122A and variants (~25 µg, see preparation in ‘FAM122A purification’ in Methods) were mixed with Expi293F whole-cell extracts expressing B55, PP2Ac constructs and purified PP2Aa. Input samples were collected prior to incubation with agarose beads. GFP-tagged B55 and associated proteins were captured by incubating equal amounts of total protein (~500 µg) for each condition with GFP-Trap nanobody agarose beads (prepared as described in ‘eGFP–nanobody protein expression, purification, and immobilization onto agarose beads’ in Methods) at 4 °C for 16 h. Following 3 washes with wash buffer (20 mM Tris pH 8.0, 500 mM NaCl, 0.5 mM TCEP, 1 mM MnCl_2_), bound proteins were eluted with 2% SDS sample buffer (90 °C, 10 min), resolved by SDS–PAGE (Bio-Rad) and transferred to PVDF membrane for western blot analysis using indicated antibodies (see Reporting summary) anti-B55 (2290 S, 1:1,000), anti-PP2Ac (MABE1783, 1:1,000), goat anti-rabbi IgG, (12005869, 1:3,000) and goat anti-mouse IgG (12004158, 1:3,000). Antibody fluorescence signals were captured using a ChemiDoc MP Imaging System (Image Lab Touch Software 2.4; Bio-Rad) and band intensities were quantified using ImageJ 1.53t. Uncropped blots are shown in Supplementary Fig. 2.

### FAM122A and ARPP19 competition assay

Purified FAM122A_Nterm_ (~25 µg) and S62 tpARPP19_S104A_ (~25 µg or 125 µg, see preparation in ‘Phosphorylation of ARPP19’ in Methods) alone or in combination were mixed with Expi293F whole-cell extracts expressing B55, PP2Ac constructs and purified PP2Aa. Input samples were collected prior to incubation with agarose beads. GFP-tagged B55 and associated proteins were captured by incubating equal amounts of total protein (500 µg) for each condition with GFP-Trap nanobody agarose beads (prepared using AminoLink Plus Immobilization Kit; ThermoFisher) at 4 °C for 16 h. Following 3 washes with wash buffer (20 mM Tris pH 8.0, 500 mM NaCl, 0.5 mM TCEP, 1 mM MnCl_2_), bound proteins were eluted with 2% SDS sample buffer (90 °C, 10 min), resolved by SDS–PAGE (Bio-Rad) and transferred to PVDF membrane for western blot analysis using indicated antibodies (see Reporting summary) anti-FAM122A (MA5-24510, 1:1,000), anti-ARPP19 (Proteintech, 11678-1-AP, 1:1,000). Antibody fluorescence signals were captured using a ChemiDoc MP Imaging System (Image Lab Touch Software 2.4; Bio-Rad) and band intensities quantified using ImageJ 1.53t. Uncropped blots shown in Supplementary Fig. 2.

### Alkaline treatment for PP2Ac methylation

For alkaline treatment, 100 μl PP2A:B55 triple complex fraction from anion exchange was mixed with NaOH to a final concentration of 0.2 M and incubated for 10 min at room temperature. The reaction was neutralized by adding HCl to a final concentration of 0.2 M and diluted to 200 μl with lysis buffer. The control reaction was treated with pre-neutralization solution (0.2 M NaOH and 0.2 M HCl) and diluted to 200 μl with lysis buffer. The samples were boiled with 2% SDS sample buffer (90 °C, 10 min), resolved by SDS–PAGE (Bio-Rad) and transferred to PVDF membrane for western blot analysis using indicated antibodies (see Reporting summary) anti-PP2Ac (MABE1783, 1:1,000), anti-PP2Ac Methyl (Leu309) (828801, 1:1,000). Antibody fluorescence signals were captured using a ChemiDoc MP Imaging System (Image Lab Touch Software 2.4; Bio-Rad) and band intensities quantified using ImageJ 1.53t. Uncropped blots shown in Supplementary Fig. 1.

### eGFP–nanobody protein expression, purification, and immobilization onto agarose beads

For expression, pOPIN-eGFP-nanobody plasmid DNA (a gift from M. Bollen) was transformed into *E. coli* BL21 (DE3) cells (Agilent). Freshly transformed cells were grown at 37 °C in LB broth containing ampicillin antibiotics (50 µg ml^−1^) until they reached an optical density (OD_600_) of ~0.8. Protein expression was induced by addition of 0.5 mM β-d-thiogalactopyranoside (IPTG) to the culture medium, and cultures were allowed to grow overnight (18–20 h, 250 rpm shaking) at 18 °C. Cells were collected by centrifugation (8,000*g*, 15 min, 4 °C) and stored at −80 °C until purification. Cell pellets expressing eGFP–nanobody were resuspended in ice-cold lysis buffer (50 mM Tris pH 8.0, 500 mM NaCl, 5 mM imidazole, 0.1% Triton X-100, EDTA-free protease inhibitor tablet (ThermoFisher)), lysed by high-pressure cell homogenization (Avestin Emulsiflex C3). Cell debris was pelleted by centrifugation (42,000*g*, 45 min, 4 °C), and the supernatant was filtered with 0.22-µm syringe filters. The proteins were loaded onto a HisTrap HP column (Cytiva) pre-equilibrated with buffer A (50 mM Tris pH 8.0, 500 mM NaCl, 5 mM imidazole) and eluted using a linear gradient (0–60% B) with buffer B (50 mM Tris pH 8.0, 500 mM NaCl and 500 mM imidazole). Fractions containing the protein were pooled, concentrated, and further purified at room temperature using SEC (Superdex 75 26/60 (Cytiva)) in PBS pH 7.5 buffer. Purified and concentrated eGFP–nanobody protein was immobilized onto agarose beads (20 mg protein per column) using AminoLink Plus Immobilization Kit (ThermoFisher), following manufacturer’s instructions in PBS pH 7.5 coupling buffer.

### B55 and B55_LL_ purification

Pellets of Expi293F cells expressing eGFP–B55 or eGFP–B55_LL_ were resuspended in ice-cold lysis buffer (20 mM Tris pH 8.0, 500 mM NaCl, 0.5 mM TCEP, 0.1% Triton X-100, EDTA-free protease inhibitor tablet (ThermoFisher)), lysed by high-pressure cell homogenization (Avestin Emulsiflex C3). Cell debris was pelleted by centrifugation (42,000*g*, 45 min, 4 °C), and the supernatant was filtered with 0.22-µm syringe filters. Lysates were mixed with GFP–nanobody-coupled agarose beads (see preparation in ‘eGFP–nanobody protein expression, purification, and immobilization onto agarose beads’ in Methods), pre-equilibrated with wash buffer 1 (20 mM Tris pH 8.0, 500 mM NaCl and 0.5 mM TCEP) and slowly rocked at 4 °C for 2 h. After 2 h, lysate–bead mixture was loaded onto gravity columns, the flow through (FT1) was collected and the column was washed 3 times with 25 ml of wash buffer (washes 1–3). The GFP–B55 resin was resuspended in 20 mM Tris pH 8.0, 250 mM NaCl and 0.5 mM TCEP, and TEV was added for on-column cleavage with rocking overnight at 4 °C. The flow through was again collected (FT2) and the resin was washed with 20 ml of wash buffer 2 (20 mM Tris pH 8.0, 250 mM NaCl and 0.5 mM TCEP; wash 4) and 2× 20 ml with the wash buffer 1 (washes 5 and 6). The flow through 2 (FT2) and washes 4–6 were collected, diluted to ~100 mM salt concentration (with 0 mM NaCl wash buffer), and loaded onto QTrap HP column (Cytiva) for further purification. The proteins were eluted with a 100 mM–1 M salt gradient (buffer A: 20 mM Tris pH 8.0, 100 mM NaCl, 0.5 mM TCEP; buffer B: 20 mM Tris pH 8.0, 1 M NaCl, 0.5 mM TCEP). B55 or B55_LL_ were concentrated and further purified using SEC (Superdex 200 26/60 (Cytiva)) in NMR buffer (20 mM Na_2_HPO_4_/NaH_2_PO_4_ pH 6.3, 150 mM NaCl, 0.5 mM TCEP) or assay buffer (20 mM Tris pH 8.0, 150 mM NaCl, 0.5 mM TCEP).

### PP2A:B55 complex purification

Expi293F cell pellets expressing StrepII–PP2Ac and eGFP–B55 constructs were resuspended in ice-cold lysis buffer (20 mM Tris pH 8.0, 500 mM NaCl, 0.5 mM TCEP, 1 mM MnCl_2_, 0.1% Triton X-100, EDTA-free protease inhibitor tablet (ThermoFisher)), lysed by high-pressure cell homogenization (Avestin Emulsiflex C3). Purified PP2Aa was added to the cell lysate. Cell debris was pelleted by centrifugation (42,000*g*, 45 min, 4 °C), and the supernatant was filtered with 0.22-µm syringe filters. Lysates were loaded onto a GFP–nanobody-coupled agarose bead (see preparation in ‘eGFP–nanobody protein expression, purification, and immobilization onto agarose beads’ in Methods) column, pre-equilibrated with wash buffer 1 (20 mM Tris pH 8.0, 500 mM NaCl, 1 mM MnCl_2_ and 0.5 mM TCEP) and slowly rocked at 4 °C for 2 h. After 2 h, the flow through (FT1) was collected and the column was washed 3 times with 25 ml of wash buffer (washes 1–3). The GFP–B55 resin was resuspended in 20 mM Tris pH 8.0, 250 mM NaCl, 1 mM MnCl_2_ and 0.5 mM TCEP, and TEV was added for on-column cleavage rocking overnight at 4 °C. The flow through was again collected (FT2) and the resin was washed with 20 ml of wash buffer 2 (20 mM Tris pH 8.0, 250 mM NaCl, 1 mM MnCl_2_ and 0.5 mM TCEP) (wash 4) and 2× 20 ml with the wash buffer 1 (washes 5 and 6). The flow through 2 (FT2) and washes 4–6 were collected, diluted to ~100 mM salt concentration (with 0 mM NaCl Wash buffer), and loaded onto Mono Q column (Cytiva) for further purification. The proteins were eluted with a 100 mM–1 M salt gradient (buffer A: 20 mM Tris pH 8.0, 100 mM NaCl, 1 mM MnCl_2_ and 0.5 mM TCEP; buffer B: 20 mM Tris pH 8.0, 1 M NaCl, 1 mM MnCl_2_ and 0.5 mM TCEP). PP2A:B55 complex and B55 fractions were pooled, concentrated and further purified using SEC (Superdex 200 26/60 (Cytiva)) in NMR buffer (20 mM Na_2_HPO_4_/NaH_2_PO_4_ pH 6.3, 150 mM NaCl and 0.5 mM TCEP) or assay buffer (20 mM Tris pH 8.0, 150 mM NaCl, 1 mM MnCl_2_ and 0.5 mM TCEP).

### Cryo-EM data acquisition and processing

The PP2A:B55–FAM122A complex was prepared by purifying PP2A:B55 and incubating it with a 1.5 molar ratio of PP2A:B55 to FAM122A_ID_ at a total concentration of 1.2 mg ml^−1^. The PP2A:B55–tpARPP19 complex was prepared by purifying PP2A:B55 and incubating it with a 1.5 molar ratio of PP2A:B55 to tpARPP19 at a total concentration of 2.4 mg ml^−1^. Immediately prior to blotting and vitrification (Vitrobot MK IV, 18 °C, 100% relative humidity, blot time 5 s), CHAPSO (3-([3-cholamidopropyl]dimethylammonio)-2-hydroxy-1-propanesulfonate) was added to a final concentration of 0.075% (w/v) for PP2A:B55-FAM122A and 0.125% (w/v) for PP2A:B55–tpARPP19. 3.5 μl of the sample was applied to a freshly glow discharged UltAuFoil 1.2/1.3 300 mesh grid, blotted for 5 s and plunged into liquid ethane. Imaging was performed using a Titan Krios G3i equipped with a Gatan BioQuantum K3 energy filter and camera operating in CDS mode. Acquisition and imaging parameters are given in Supplementary Table 1. All data processing steps were performed using Relion 4.0^53^ and are summarized in Extended Data Figs. 6–8. For both datasets, micrograph movies were summed and dose-weighted; contrast transfer function (CTF) parameters were estimated using CTFFind 4.1.14^54^ on movie frame-averaged power spectra (~4 e Å^−2^ dose). Micrographs were filtered to remove outliers in motion correction and/or CTF estimation results and screened manually to remove micrographs with significant non-vitreous ice contamination. Potential particle locations on the full micrograph set were selected using Topaz^55^ using a model trained on a random subset of the micrographs. Particles on the training subset were selected by a Topaz model trained on previous screening data. Subset picks were subjected to 2D classification, ab initio 3D initial model generation, and 3D classification, and surviving particles used to train an improved Topaz model used to pick the full micrograph set. From these picks, 2D classification and 3D classification (with full angular and translational searches) were used to select particles in classes showing clear secondary structure and representing the full complex. Resolution in both datasets was then further improved by cycles of CTF parameter refinement, particle polishing, and fixed-pose 3D classification, alongside the following elaborations: For PP2A:B55–tpARPP19, particles with well-resolved ARPP19 density were selected by isolating ARPP19 via signal subtraction of the vast majority of the holoenzyme, followed by fixed-pose 3D classification; this process was performed twice in the course of the processing workflow. The final map was refined from 52,934 particles to a resolution of 2.77 Å. For PP2A:B55–FAM122A, multi-body refinement of the B55 and PP2Ac segments of the complex was needed to resolve details of both segments. Within each resulting body alignment, signal subtraction and fixed-pose 3D classification of FAM122A and its surrounding binding groove was used to select for particles for which multi-body refinement was successful and FAM122A was present and well-resolved. This yielded 103,522 particles for which this was simultaneously true in both bodies. Using these particles, a second multi-body refinement was used to generate maps for model building within each body, with final resolutions of 2.55 Å for the B55 body and 2.69 Å for the PP2Ac body. To generate a consensus map, a refinement was run using only the top 25,000 particles with the smallest sum of squared eigenvalues from the multi-body refinement (as reported by relion_flex_analyse). All 3D auto-refinements for both datasets utilized a soft solvent mask and SIDESPLITTER^56^. All global map resolutions reported in this work were calculated by the gold-standard half-maps Fourier shell correlation (FSC) = 0.143 metric. Further validation information is given in Extended Data Figs. 6–8 and Supplementary Table 1.

### Cryo-EM model building

All models were built and refined by iterating between manual rebuilding and refinement in Coot^57^ and ISOLDE^58^, and automated global real-space refinement in Phenix^59^. For PP2A:B55–FAM122A, the relevant segments of the model were built into the B55 and PP2Ac body maps, using the previously determined crystal PP2A:B55 holoenzyme crystal structure (PDB ID 3DW8) and the available FAM122A AlphaFold model (UniProt Q96E09) as a starting point. The two body models were then joined, and the regions near the joints further rebuilt, and the entire complex refined against the 25,000-particle consensus subset map. For PP2A:B55–tpARPP19, the holoenzyme portion of the PP2A:B55-FAM122A model and the available ARPP19 AlphaFold model (UniProt P56211) were used as starting points. Model geometry and map–model validation metrics are given in Supplementary Table 1. Maps in Fig. 2 are LAFTER filtered and sharpened maps^60^.

### PP2A:B55 activity assay

Phosphatase activity assays were conducted in 96 well plates (Corning). PP2A:B55 holoenzyme was diluted to desired concentration range (0 to 20 nM) in Enzyme buffer (30 mM HEPES pH 7.0, 150 mM NaCl, 1 mM MnCl_2_, 1 mM DTT, 0.01% Triton X-100, 0.1 mg ml^−1^ BSA) and incubated at 30 °C. The reaction was started by the addition of 6,8-difluoro 4-methylumbelliferyl phosphate (DiFMUP) to a final concentration of 50 μM. Assays were read every 15 s for ~50 min on a CLARIOstarPlus (BMG LABTECH) plate reader (using reader control software v. 5.7 R2) and the data was evaluated using GraphPad Prism 9.5.

### DiFMUP fluorescence intensity assay for PP2A:B55 IC_50_ measurements

DiFMUP based IC_50_ assays were conducted in 384-well plates (Corning, 4411). For ARPP19 and FAM122A IC_50_ assays, PP2A:B55 holoenzyme in Enzyme buffer (30 mM HEPES pH 7.0, 150 mM NaCl, 1 mM MnCl_2_, 1 mM DTT, 0.01% triton X-100, 0.1 mg ml^−1^ BSA) was pre-incubated with various concentrations of ARPP19 and FAM122A variants for 30 min at room temperature (Extended Data Fig. 2). The reaction was started by adding DiFMUP (final concentration 50 μM) into the PP2A:B55-FAM122A enzymatic reaction (final concentration of PP2A:B55 holoenzyme at 1 nM) and then incubated at 30 °C for 30 min. End-point reads (excitation 360 nm, emission 450 nm) were taken on a CLARIOstarPlus (BMG LABTECH) plate reader (using reader control software version 5.7 R2) after the reaction was stopped by the addition of 300 mM potassium phosphate (pH 10). The experiments were independently repeated ≥ 3 times (each reaction was made in *n* = 3 to 6) and the averaged IC_50_ and s.d. values were reported. The data was evaluated using GraphPad Prism 9.5.

### Fluorescence polarization PP2A binding assays

Following the instructions of the manufacturer, 100 µM of FAM122A_ID_(S120C) (or variants) or ARPP19(S10C) was labelled with Alexa Fluor 488 C5 Maleimide (ThermoFisher) using 1:10 protein to fluorophore ratio. The mixture was incubated for 2 h in the dark at room temperature at pH 7.0 and excess β-mercaptoethanol (1.2× the concentration of the fluorophore) was added to inactivate any unreacted Alexa Fluor 488. Labelled FAM122A_ID_(S120C) (or variants) or ARPP19(S10C) was recovered by analytical SEC (Superdex 75 Increase 10/300 (Cytiva)) and used for the fluorescence polarization assays. The labelled FAM122A_ID_(S120C) (or variants) or ARPP19(S10C) are hereafter referred to as FAM122A_ID_-tracer, or ARPP19-tracer.

The fluorescence polarization assays were standardized using black 384-well low volume round bottom microplates (Corning, 4411) with 15 µl solution per well. The measurements were performed using a CLARIOstarPlus (BMG LABTECH Inc) microplate reader (using reader control software version 5.7 R2) set up to 482 ± 16 nm excitation, 530 ± 40 nm emission, and dichroic long pass filter 504 nm with reflection ranging between 380–497 nm and transmission ranging between 508–850 nm. For the dissociation constant (*K*_d_) binding measurements, all dilutions were made into fluorescence polarization buffer (10 mM HEPES pH 7.0, 150 mM NaCl, 0.5 mM TCEP, 0.01% Triton X-100, 0.1 mg ml^−1^ BSA). A predilution of FAM122A_ID_-tracer/ARPP19-tracer was prepared for 0.3 nM and a serial dilution of PP2A:B55 was made at 3 times the final concentration. Five microlitres of FAM122A_ID_-tracer/ARPP19-tracer, 5 µl of serially diluted PP2A:B55 complex and 5 µl of fluorescence polarization buffer were distributed into the 384-well microplate, resulting in a 0.1 nM final concentration of FAM122A_ID_-tracer or ARPP19-tracer. All assay experiments were repeated in triplicate and incubated for 30 min in the dark and sealed at room temperature before reading. The experiments were independently repeated ≥3 times and the averaged *K*_d_ and s.d. values were reported. The data was evaluated using GraphPad Prism 9.5.

### ARPP19 immunoprecipitation

Synthetic DNA encoding the various ARPP19 sequences was purchased from GeneArt, Life Technologies and cloned into the pcDNA5/FRT/TO (Invitrogen) expression vector containing YFP resulting in YFP–ARPP19 fusion proteins. These constructs were transiently transfected into HeLa cells 24 h prior to collecting cells. Cells were lysed in lysis buffer (50 mM Tris-HCl pH 7.5, 50 mM NaCl, 1 mM EDTA, 1 mM DTT and 0.1% NP40). Complexes were immunoprecipitated at 4 °C in lysis buffer with GFP-Trap (ChromoTek) beads as described by the manufacturer. Precipitated protein complexes were washed 3 times in lysis buffer, eluted in 2× SDS sample buffer and subjected to western blotting using the following antibodies: YFP (1:5,000; generated in house), B55α (1:2,000; 5689S, Cell Signaling Technology), PP2Ac (1:2,000; 05-421, Millipore). Uncropped blots are shown in Supplementary Fig. 1.

### NMR data collection

All NMR data were collected on either a Bruker Avance Neo 600 MHz or 800 MHz NMR spectrometer equipped with TCI HCN *z*-gradient cryoprobe at 283 K. (^15^N,^13^C)-labelled FAM122A_Nterm_ (150 µM), (^15^N,^13^C)-labelled FAM122A_ID_ (400 µM), (^15^N,^13^C)-labelled ARPP19 (400 µM) and (^15^N,^13^C)-labelled *pS62pS104*ARPP19 (200 µM) were prepared in either FAM122A or ARPP19 NMR buffer with 5-10% (v/v) D_2_O added immediately prior to data acquisition. The sequence-specific backbone assignments both proteins were determined by recording a suite of heteronuclear NMR spectra: 2D ^1^H,^15^N HSQC, 3D HNCA, 3D HN(CO)CA, 3D HNCACB, 3D CBCA(CO)NH, 3D HNCO, and 3D HN(CA)CO, with an additional spectrum, 3D (H)CC(CO)NH, collected for FAM122A_ID_ (*t*_m_ = 12 ms)^61^. Spectra were processed in Topspin (Bruker Topspin 4.1.3) and referenced to internal DSS.

### Sequence-specific backbone assignment, chemical shift index and chemical shift perturbation

Peak picking and sequence-specific backbone assignment were performed using CARA 1.9.1 (http://www.cara.nmr.ch). CSI calculations of FAM122A_Nterm_, FAM122A_ID_, ARPP19 and pS62pS104ARPP19 were performed using both Cα and Cβ chemical shifts for each assigned amino acid, omitting glycine, against the RefDB database^62^. Secondary structure propensity (SSP) scores were calculated using a weighted average of seven residues to minimize contributions from chemical shifts of residues that are poor measures of secondary structure^63^. The changes in peak position between different FAM122A or ARPP19 constructs or variants were traced according to nearest neighbour analysis. Chemical shift differences (∆*δ*) were calculated using the following equation:

$$\Delta {\delta }{\rm{(ppm)}}=\sqrt{{(\varDelta {{\delta }}_{{\rm{H}}})}^{2}+{(\varDelta {{\delta }}_{{\rm{N}}}/5)}^{2}}$$

### NMR interaction studies of FAM122A and ARPP19 with PP2A:B55 and B55_LL_

All NMR interaction data of FAM122A_Nterm/ID_, ARPP19 or pS62pS104ARPP19 with either PP2A:B55 or B55_LL_ were recorded using a Bruker Neo 600 MHz NMR spectrometer equipped with a HCN TCI active *z*-gradient cryoprobe at 283 K. All NMR measurements of FAM122A_Nterm_ or FAM122A_ID_ or ARPP19 and pS62pS104ARPP19 were recorded using ^15^N-labelled protein in NMR buffer and 90% H_2_O/10% D_2_O. For each interaction, an excess of unlabelled B55_LL_ of PP2A:B55 complex (min 25% surplus ratio) was added to the ^15^N-labelled FAM122A or ARPP19 construct under investigation and incubated on ice for 10 min before the 2D ^1^H,^15^N HSQC spectrum was collected. FAM122A and ARPP19 concentrations ranged from 2–6 μM. NMR data were processed using nmrPipe^64^ and the intensity data were analysed in Poky^65^. Each dataset was normalized to its respective most intense peak and the difference between each free 2D ^1^H,^15^N HSQC spectrum FAM122A or ARPP19 residue was compared to its respective peak, if present, on the 2D ^1^H,^15^N HSQC spectrum of FAM122A or ARPP19 in complex with B55_LL_ or PP2A:B55. Any overlapping peaks were omitted for this analysis.

### Reporting summary

Further information on research design is available in the Nature Portfolio Reporting Summary linked to this article.

## Online content

Any methods, additional references, Nature Portfolio reporting summaries, source data, extended data, supplementary information, acknowledgements, peer review information; details of author contributions and competing interests; and statements of data and code availability are available at 10.1038/s41586-023-06870-3.

### Supplementary information

Supplementary Information This file contains Supplementary Figs. 1 and 2 and Supplementary Table 1.

Reporting Summary

## Figures and Tables

**Extended Data Table 1:**
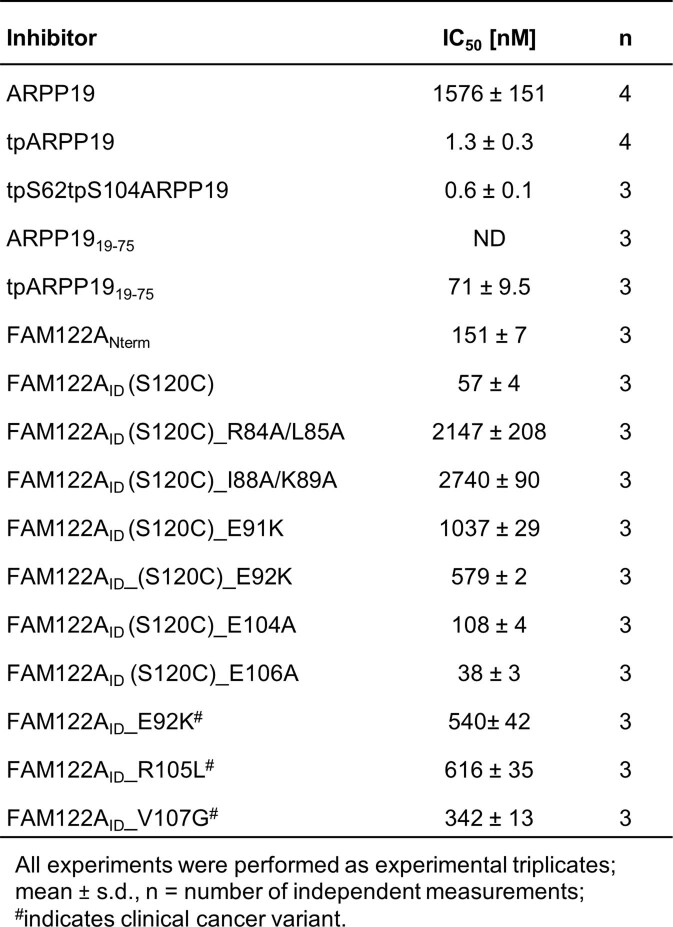
Inhibition (IC_50_) of PP2A:B55 by ARPP19 and FAM122A

Table reporting the inhibition of the PP2A:B55 holoenzyme by ARPP19 and FAM122A and its variants; tp, thiophosphorylation; ^#^clinical cancer variants.

**Extended Data Table 2:**
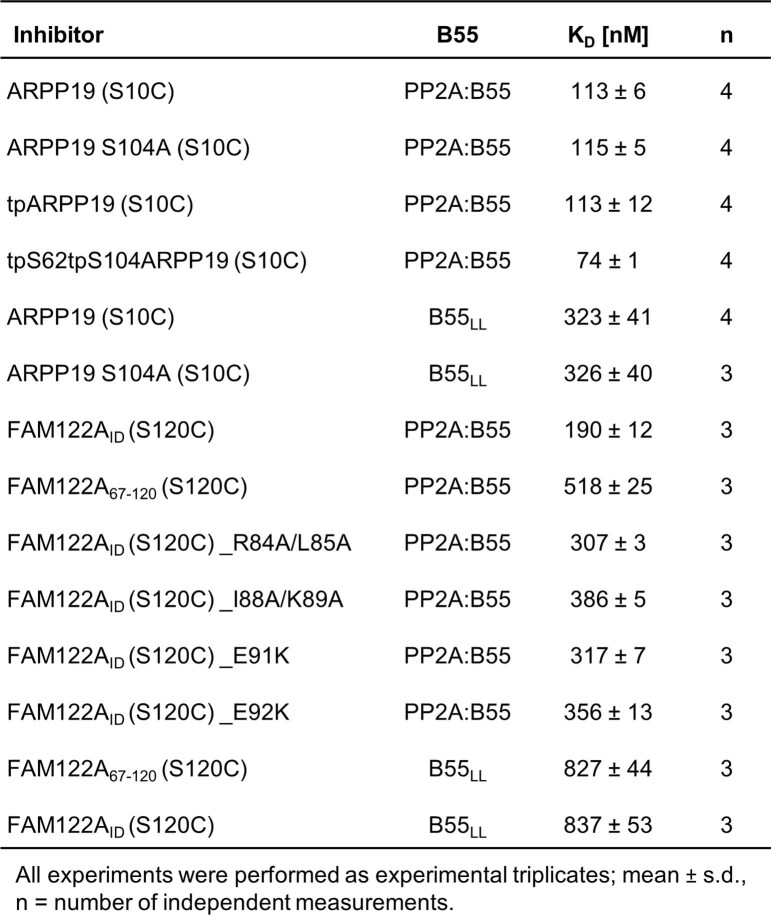
Binding affinity (K_D_) of ARPP19 and FAM122A to PP2A:B55 or B55_LL_

Table reporting the binding affinity of the PP2A:B55 holoenzyme or B55_LL_ vs ARPP19 and FAM122A and its variants; tp, thiophosphorylation.

## Data Availability

The NMR data generated in this study have been deposited in the BioMagResBank database under accession codes BMRB 51828 (FAM122A_Nterm_), 51682 (FAM122A_ID_), 51881 (ARPP19) and 51882 (tpS62tpS104ARPP19). The atomic coordinates and structure factors for PP2A:B55–tpARPP19 complex have been deposited in the PDB database under accession code 8TTB and EMDB code EMD-41604. The atomic coordinates and structure factors for PP2A:B55-FAM122A complex have been deposited in the PDB database under accession code 8SO0 (10.2210/pdb8so0/pdb) and EMDB code EMD-40644 (B55 body, 8TWE/EMD-41667; catalytic body, 8TWI, EMD-41668). All IC_50_, fluorescence polarization and pull-down data generated in this study are provided in the Supplementary Information and/or Source Data file, which is available at Figshare (10.6084/m9.figshare.23992656).
