# Supplementary material for: Cryo-EM structures of PP2A:B55–FAM122A and PP2A:B55–ARPP19
Source: Nature. 2023 Dec 20;625(7993):195–203. doi: 10.1038/s41586-023-06870-3 (PMC10765524; doi:10.1038/s41586-023-06870-3)
Supplement: Supplementary file 1 — This file contains Supplementary Figs. 1 and 2 and Supplementary Table 1. [file 41586_2023_6870_MOESM1_ESM.pdf]

nature portfolio
https://doi.org/10.1038/s41586-023-06870-3

Supplementary information

Cryo-EM structures of PP2A:B55–FAM122A and PP2A:B55–ARPP19

In the format provided by the
authors and unedited

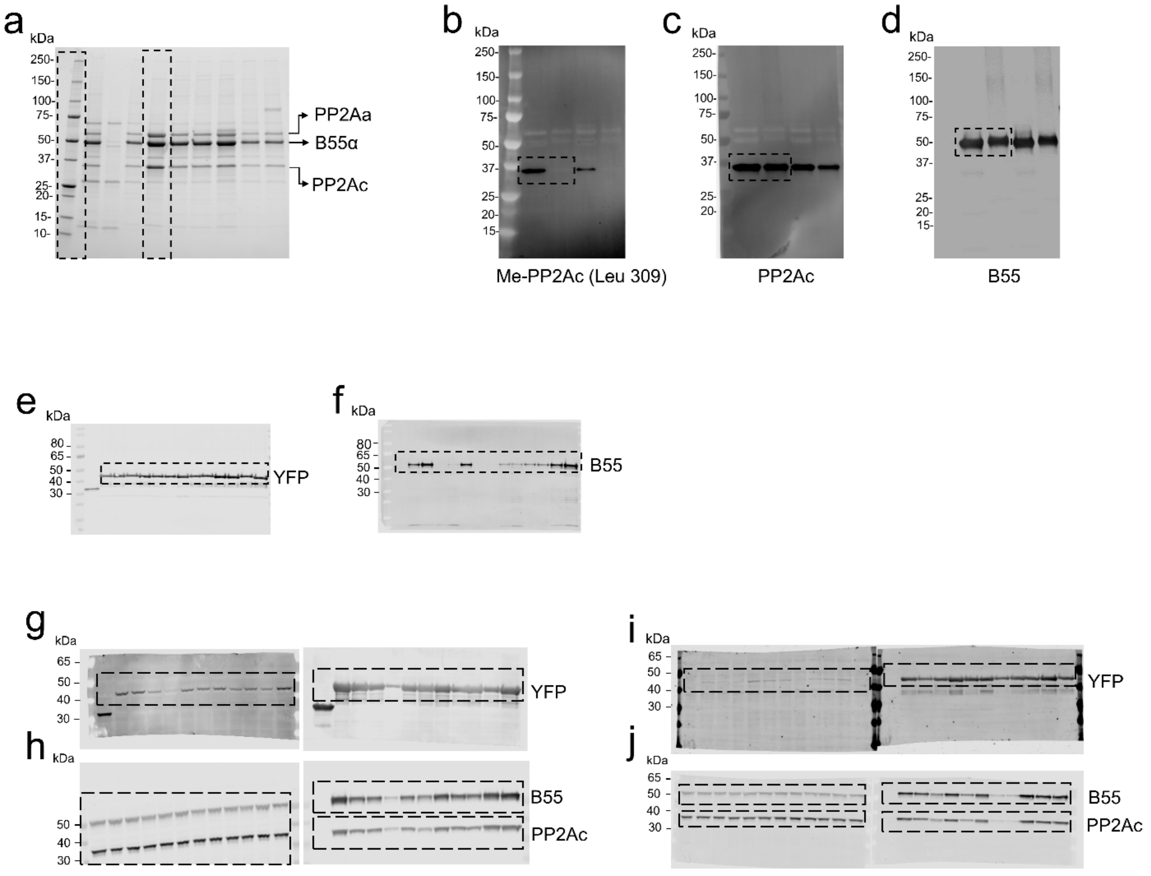

Supplementary Fig. 1. Uncropped western blot images. a-d. **Refer to Extended Data Fig. 1c.** See legend and panel in Extended Data Fig. 1c for lane identifiers. Proteins were detected by western blotting, using the indicated antibodies. Bands shown in Extended Data Fig. 1c are indicated by a dashed line box. **e-f**. **Refer to Fig. 3b.** See legend and panel in Fig. 3b for lane identifiers. Proteins were detected by western blotting, using the indicated antibodies. Bands shown in Fig. 3b are indicated by a dashed line box. **g-j. Refers to Fig. 3e and Extended Data Fig. 9e-g.** See legend and panel in Fig. 3e and Extended Data Fig. 9e-g for lane identifiers. Proteins were detected by western blotting, using the indicated antibodies. Bands shown in Fig. 3e and Extended Data Fig. 9e-g are indicated by a dashed line box. Molecular weight markers (kDa) shown.

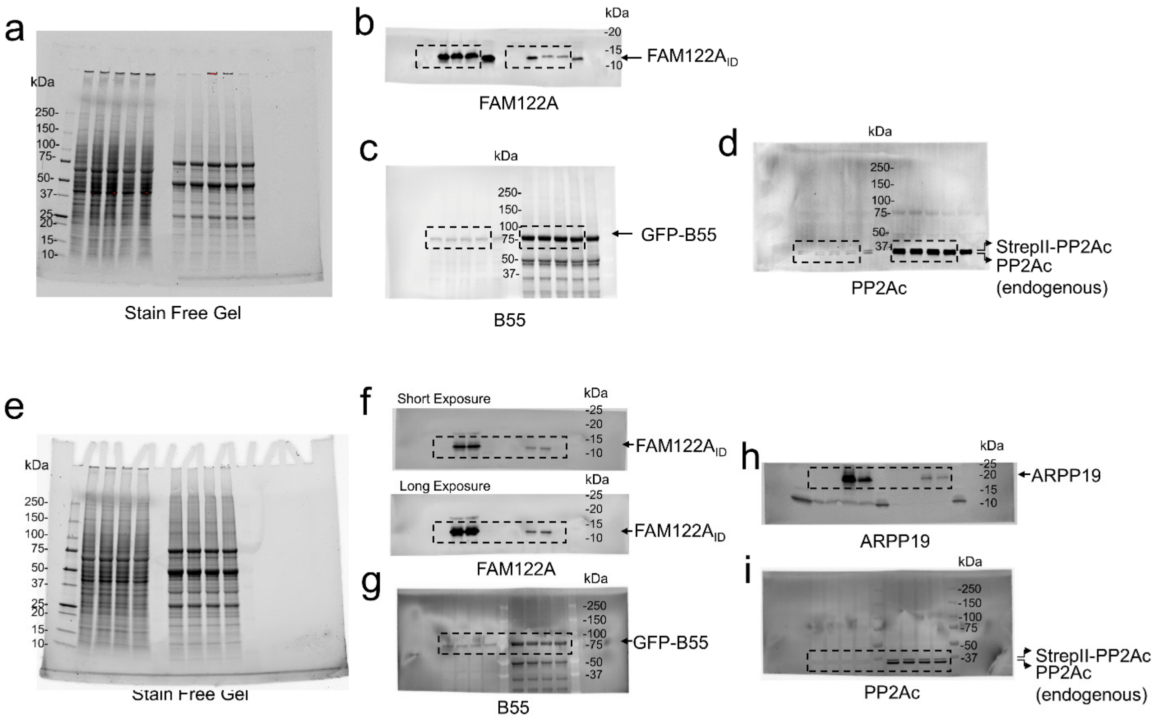

Supplementary Fig. 2. Uncropped gel and western blot images. a-d. Refers to Fig 4h. Stain-free SDS-PAGE gel (**a**) used for Western blots (**b-d)**. See legend and panel in 4h for lane identifiers. Proteins were detected by western blotting, using the indicated antibodies. Bands shown in Fig. 4h indicated by a dashed line box. **e-i**. **Refers to Extended Data Fig. 10d**. Stain-free SDS-PAGE (**e**) used for Western blots (**f-i**). See legend and panel in Extended Data Fig. 13d for lane identifiers. Proteins were detected by western blotting, using the indicated antibodies. Bands shown in Extended Data Fig. 10d indicated by a dashed line box. Molecular weight markers (kDa) shown.

Supplementary Table 1. Cryo-EM data collection, refinement and validation statistics

	PP2A:B55-FAM122A			
	**B55 body (EMDB: EMD-41667) (PDB: 8TWE)**	**Catalytic body (EMDB: EMD-41668) (PDB: 8TWI)**	**Consensus (EMDB: EMD-40644) (PDB: 8SO0)**	**PP2A:B55-*tp*ARPP19 (EMDB: EMD-41604) (PDB: 8TTB)**
**Data collection and processing**				
Magnification	81,000x	81,000x	81,000x	105,000x
Voltage (kV)	300	300	300	300
Electron exposure (e–/Å^2)	70	70	70	70
Defocus range (μm)	-2.53 to -0.55	-2.53 to -0.55	-2.33 to -0.56	-2.60 to -0.40
Pixel size (Å)	1.068	1.068	1.068	0.827
Symmetry imposed	C1	C1	C1	C1
Initial particle images (no.)	1,248,538	1,248,538	1,248,538	1,170,216
Final particle images (no.)	103,522	103,522	25,000	52,934
Map resolution (Å)<br>FSC threshold	2.55<br>FSC = 0.143	2.69<br>FSC = 0.143	2.80<br>FSC = 0.143	2.77<br>FSC = 0.143
Map resolution range (Å)	4.58 to 2.48	4.02 to 2.68	5.96 to 2.75	5.14 to 2.71
**Refinement**				
Initial model used (PDB)	3DW8			3DW8
Model resolution (Å)<br>FSC threshold	2.61<br>FSC = 0.5	3.12<br>FSC = 0.5	2.86<br>FSC = 0.5	2.81<br>FSC = 0.5
Map sharpening *B* factor (Å^2)	-52.965	-71.906	-32.336	-57.838
Model composition<br>Non-hydrogen atoms<br>Protein residues<br>Ligands	 6683<br>833<br>None	 4073<br>510<br>ZN:1<br>FE:1	 10756<br>1343<br>ZN:1<br>FE:1	 11002<br>1376<br>ZN:1<br>FE:1
B factors (Å^2) (min/max/mean)<br>Protein<br>Ligand	 27.60/179.51/ 89.51<br>N/A	 33.76/188.61/ 121.80<br>162.24/187.03/ 174.63	 8.88/155.34/ 78.91<br>113.84/135.82/ 124.83	 41.46/188.26/ 86.61<br>98.59/113.27/ 105.93
R.m.s. deviations<br>Bond lengths (Å)<br>Bond angles (°)	 0.003<br>0.463	 0.002<br>0.460	 0.002<br>0.400	 0.002<br>0.398
Validation<br>MolProbity score<br>Clashscore<br>Poor rotamers (%)	 0.81<br>1.05<br>0	 0.88<br>1.24<br>0	 0.95<br>1.87<br>0	 1.15<br>3.65<br>0
Ramachandran plot<br>Favored (%)<br>Allowed (%)<br>Disallowed (%)	 99.51<br>0.49<br>0	 97.82<br>2.18<br>0	 98.11<br>1.89<br>0	 98.30<br>1.70<br>0
