# Supplementary material for: Cryo-EM structures of PP2A:B55–FAM122A and PP2A:B55–ARPP19
Source: Nature. 2023 Dec 20;625(7993):195–203. doi: 10.1038/s41586-023-06870-3 (PMC10765524; doi:10.1038/s41586-023-06870-3)
Supplement: Supplementary file 2 — Reporting Summary [file 41586_2023_6870_MOESM2_ESM.pdf]

nature portfolio

Corresponding author(s): Wolfgang Peti
Last updated by author(s): Oct 26, 2023

Reporting Summary

Nature Portfolio wishes to improve the reproducibility of the work that we publish. This form provides structure for consistency and transparency in reporting. For further information on Nature Portfolio policies, see our Editorial Policies and the Editorial Policy Checklist.

Statistics

For all statistical analyses, confirm that the following items are present in the figure legend, table legend, main text, or Methods section.

n/a	Confirmed	
☐	☒	The exact sample size (*n*) for each experimental group/condition, given as a discrete number and unit of measurement
☐	☒	A statement on whether measurements were taken from distinct samples or whether the same sample was measured repeatedly
☒	☐	The statistical test(s) used AND whether they are one- or two-sided<br>*Only common tests should be described solely by name; describe more complex techniques in the Methods section.*
☒	☐	A description of all covariates tested
☐	☒	A description of any assumptions or corrections, such as tests of normality and adjustment for multiple comparisons
☐	☒	A full description of the statistical parameters including central tendency (e.g. means) or other basic estimates (e.g. regression coefficient) AND variation (e.g. standard deviation) or associated estimates of uncertainty (e.g. confidence intervals)
☒	☐	For null hypothesis testing, the test statistic (e.g. *F*, *t*, *r*) with confidence intervals, effect sizes, degrees of freedom and *P* value noted<br>*Give P values as exact values whenever suitable.*
☒	☐	For Bayesian analysis, information on the choice of priors and Markov chain Monte Carlo settings
☒	☐	For hierarchical and complex designs, identification of the appropriate level for tests and full reporting of outcomes
☒	☐	Estimates of effect sizes (e.g. Cohen's *d*, Pearson's *r*), indicating how they were calculated

Our web collection on statistics for biologists contains articles on many of the points above.

Software and code

Policy information about availability of computer code

Data collection	Bruker Topspin 4.1.3, Clariostar Mars 3.42, SerialEM 3.8, Image Lab Touch Software 2.4
Data analysis	GraphPad Prism 9.5, CARA 1.8.4.2, CcPNMR 2.5.2, NMRFARM SPARKY 1.47, SigmaPlot 14.5, Microsoft® Excel® for Microsoft 365 MSO (Version 2301 Build 16.0.16026.20002) 64-bit; Relion 4, ChimeraX 1.3, Coot 0.9.2, Image Lab Touch Software 2.4, ImageJ 1.53t, Topaz 0.2, Isolde, Phenix 1.20, GraphPad Prism 9.5, Bruker Topspin 4.1.3

For manuscripts utilizing custom algorithms or software that are central to the research but not yet described in published literature, software must be made available to editors and reviewers. We strongly encourage code deposition in a community repository (e.g. GitHub). See the Nature Portfolio guidelines for submitting code & software for further information.

Data

Policy information about availability of data

All manuscripts must include a data availability statement. This statement should provide the following information, where applicable:

- Accession codes, unique identifiers, or web links for publicly available datasets
- A description of any restrictions on data availability
- For clinical datasets or third party data, please ensure that the statement adheres to our policy

BMRB: 51828, 51682, 51881, 51882; PDB: 8SO0, 8TTB, 8TWE, 8TW1; EMD-40644, EMD-41604, EMD-41667, EMD-41668. The source data underlying figures and tables are provided as a Source Data file and on Figshare (10.6084/m9.figshare.23992656)

Research involving human participants, their data, or biological material

Policy information about studies with human participants or human data. See also policy information about sex, gender (identity/presentation), and sexual orientation and race, ethnicity and racism.

Reporting on sex and gender	N/A
Reporting on race, ethnicity, or other socially relevant groupings	N/A
Population characteristics	N/A
Recruitment	N/A
Ethics oversight	N/A

Note that full information on the approval of the study protocol must also be provided in the manuscript.

Field-specific reporting

Please select the one below that is the best fit for your research. If you are not sure, read the appropriate sections before making your selection.

☒ Life sciences ☐ Behavioural & social sciences ☐ Ecological, evolutionary & environmental sciences

For a reference copy of the document with all sections, see nature.com/documents/nr-reporting-summary-flat.pdf

Life sciences study design

All studies must disclose on these points even when the disclosure is negative.

Sample size	SDS-PAGE, western blot and fluorescence based assays were done with an independent n=2 to 6 and showed to have excellent reproducibility; mean +/- STD was used for statistical analysis as commonly used for these techniques.
Data exclusions	No data was excluded in our analysis.
Replication	All data was replicated at least twice in independent studies. Data was also collected from different protein expression/purification batches, i.e.., not just technical replicates, but also experimental replicates.
Randomization	No randomization was necessary for our in vitro biochemical studies as sample bias is impossible due to the use of experimental replicates.
Blinding	Blinding was not relevant for the study as no test subjects were used in this study. Furthermore no experimental grouping or randomization is required for this work.

Reporting for specific materials, systems and methods

We require information from authors about some types of materials, experimental systems and methods used in many studies. Here, indicate whether each material, system or method listed is relevant to your study. If you are not sure if a list item applies to your research, read the appropriate section before selecting a response.

Materials & experimental systems

n/a	Involved in the study	
☐	☒	Antibodies
☐	☒	Eukaryotic cell lines
☒	☐	Palaeontology and archaeology
☒	☐	Animals and other organisms
☒	☐	Clinical data
☒	☐	Dual use research of concern
☒	☐	Plants

Methods

n/a	Involved in the study	
☒	☐	ChIP-seq
☒	☐	Flow cytometry
☒	☐	MRI-based neuroimaging

Antibodies

Antibodies used	Anti-B55α (PPP2R2A (2G9), Cell Signaling Technologies, Mouse mAb #5689, 1:2000)<br>Anti-B55α (PP2A B Subunit (100C1) Rabbit mAb, Cell Signaling Technologies, Cat # 2290S, 1:1000)

Anti-PP2Ac alpha/beta (Clone 11H12, Mouse mAb, Millipore, Cat# MABE1783, Lot# Q3046542, 1:1000)
Anti-PP2Ac (Anti-PP2A Antibody, C subunit, clone 1D6, Millipore Sigma, Cat#05-421, 1:2000)
YFP (in-house generated, 1:5000)
Anti-PPP2R1A (Clone 6G3, Biolegend, Cat# 824901, 1:1000)
Anti-PP2Ac Methyl (Leu309) (Clone 2A10, Biolegend, Cat # 828801, Lot: B349332, 1:1000)
Anti-FAM122A (Clone 3E9, ThermoFisher Scientific, Cat# MA5-24510, 1:1000)
Anti-ARPP19 (Proteintech, Cat#11678-1-AP, 1:1000)
Goat anti-Rat IgG, DyLight 800 (ThermoFisher Scientific, Cat# SA5-10024, 1:3000)
Goat anti-Mouse IgG, StarBright Blue 700 (Bio-Rad, Cat# 12004158, 1:3000)
Goat anti-Rabbi IgG, StarBright Blue 520 (Bio-Rad, Cat# 12005869, 1:3000)

Validation

All antibodies used in this study are commercially available and have been validated by the manufacturer and previous publications
Anti-B55α (https://www.cellsignal.com/products/primary-antibodies/ppp2r2a-2g9-mouse-mab/5689?_requestid=7384374 PMID: 36781846, PMID: 35695070)
Anti-B55α (https://www.cellsignal.com/products/primary-antibodies/pp2a-b-subunit-100c1-rabbit-mab/2290. PMID: 34911954, 34794320)
Anti-PP2Ac (https://www.emdmillipore.com/US/en/product/Anti-PP2AC-alpha-beta-Antibody-clone-11H12,MM_NF-MABE1783-25UL.)
Anti-PP2Ac (https://www.emdmillipore.com/US/en/product/Anti-PP2A-Antibody-C-subunit-clone-1D6,MM_NF-05-421. PMID: 26310906, PMID: 24618897)
Anti-PPP2R1A (https://www.biolegend.com/en-us/clone-search?GroupID=&PageNum=69. PMID: 9032296)
Anti-PP2Ac Methyl (Leu309) (https://www.biolegend.com/en-us/products/purified-anti-pp2ac-methyl-leu-309-antibody-11522. PMID: 31992581)
Anti-FAM122A (https://www.thermofisher.com/antibody/product/FAM122A-Antibody-clone-3E9-Monoclonal/MA5-24510. PMID: 33108758)
Anti-ARPP19 (https://www.ptglab.com/products/ARPP-19-Antibody-11678-1-AP.htm. PMID: 31717978, 32753897)
Goat anti-Rat IgG, DyLight 800 (https://www.thermofisher.com/antibody/product/Goat-anti-Rat-IgG-H-L-Cross-Adsorbed-Secondary-Antibody-Polyclonal/SA5-10024, PMID: 34376643, 36106016)
Goat anti-Mouse IgG, StarBright Blue 700 (https://www.bio-rad.com/en-us/sku/12004158-starbright-blue-700-goat-anti-mouse-igg-400-ul?ID=12004158. PMID: 36261268, 36056072)
Goat anti-Rabbi IgG, StarBright Blue 520 (https://www.bio-rad-antibodies.com/polyclonal/rabbit-lapine-igg-antibody-120058.html?f=starbright%20blue%20520. PMID: 37078570, 36261268, 34279219)
YFP antibody: bands of correct size recognized only upon expression of YFP tagged proteins. Used in Kruse et al EMBO J 2020 (PMID: 32400009) and Kruse Nat Commun 2021 (PMID: 34799561).

Eukaryotic cell lines

Policy information about cell lines and Sex and Gender in Research

Cell line source(s)	Expi293F were purchased from Thermo Scientific Cat# A14527. HeLa cells were from ATCC (CCL-2).
Authentication	No further authentication was performed
Mycoplasma contamination	Cell lines were not tested for mycoplasma contamination
Commonly misidentified lines (See ICLAC register)	No commonly misidentified cell line was used in this study.
